# Breathing Abnormalities During Sleep and Wakefulness in Rett Syndrome: Clinical Relevance and Paradoxical Relationship With Circulating Pro-oxidant Markers

**DOI:** 10.3389/fneur.2022.833239

**Published:** 2022-03-29

**Authors:** Silvia Leoncini, Cinzia Signorini, Lidia Boasiako, Valeria Scandurra, Joussef Hayek, Lucia Ciccoli, Marcello Rossi, Roberto Canitano, Claudio De Felice

**Affiliations:** ^1^Rett Syndrome Trial Center, Child Neuropsychiatry Unit, University Hospital Azienda Ospedaliera Universitaria Senese, Siena, Italy; ^2^Neonatal Intensive Care Unit, University Hospital Azienda Ospedaliera Universitaria Senese, Siena, Italy; ^3^Department of Molecular and Developmental Medicine, University of Siena, Siena, Italy; ^4^Child Neuropsychiatry Unit, University Hospital, Azienda Ospedaliera Universitaria Senese, Siena, Italy; ^5^Respiratory Pathophysiology and Rehabilitation Unit, University Hospital, Azienda Ospedaliera Universitaria Senese, Siena, Italy

**Keywords:** Rett syndrome, sleep-wakefulness cycle, respiratory dysfunction, oxidative stress, non-protein-bound iron

## Abstract

**Background:**

Breathing abnormalities are common in Rett syndrome (RTT), a pervasive neurodevelopmental disorder almost exclusively affecting females. RTT is linked to mutations in the methyl-CpG-binding protein 2 (*MeCP2*) gene. Our aim was to assess the clinical relevance of apneas during sleep-wakefulness cycle in a population with RTT and the possible impact of apneas on circulating oxidative stress markers.

**Methods:**

Female patients with a clinical diagnosis of typical RTT (*n* = 66), *MECP2* gene mutation, and apneas were enrolled (mean age: 12.5 years). Baseline clinical severity, arterial blood gas analysis, and red blood cell count were assessed. Breathing was monitored during the wakefulness and sleep states (average recording time: 13 ± 0.5 h) with a portable polygraphic screening device. According to prevalence of breath holdings, the population was categorized into the wakefulness apnea (WA) and sleep apnea (SA) groups, and apnea-hypopnea index (AHI) was calculated. The impact of respiratory events on oxidative stress was assessed by plasma and intra-erythrocyte non-protein-bound iron (P-NPBI and IE-NPBI, respectively), and plasma F_2_-isoprostane (F_2_-IsoP) assays.

**Results:**

Significant prevalence of obstructive apneas with values of AHI > 15 was present in 69.7% of the population with RTT. The group with SA showed significantly increased AHI values > 15 (*p* = 0.0032), total breath holding episodes (*p* = 0.007), and average SpO_2_ (*p* = 0.0001) as well as lower nadir SpO_2_ (*p* = 0.0004) compared with the patients with WAs. The subgroups of patients with WA and SA showed no significant differences in arterial blood gas analysis variables (*p* > 0.089). Decreased mean cell hemoglobin (MCH) (*p* = 0.038) was observed in the group with WAs. P-NPBI levels were significantly higher in the group with WA than in that with SAs (*p* = 0.0001). Stepwise multiple linear regression models showed WA being related to nadir SpO_2_, average SpO_2_, and P-NPBI (adjusted *R*^2^ = 0.613, multiple correlation coefficient = 0.795 *p* < 0.0001), and P-NPBI being related to average SpO_2_, blood PaCO_2_, red blood cell mean corpuscular volume (MCV), age, and topiramate treatment (adjusted *R*^2^ = 0.551, multiple correlation coefficient = 0.765, *p* < 0.0001).

**Conclusion:**

Our findings indicate that the impact of apneas in RTT is uneven according to the sleep-wakefulness cycle, and that plasma redox active iron represents a potential novel therapeutic target.

## Introduction

Sleep disorders are variably prevalent in rare genetic syndromes (1) such as Rett syndrome (RTT, OMIM #312750) (2, 3), a pervasive neurodevelopmental disorder affecting almost exclusively females (1:10,000) and mainly linked to mutations in the gene encoding methyl-CpG-binding protein 2 (MeCP2) (4). Although a rare disorder, RTT represents a leading cause of severe cognitive impairment in the female gender (5).

Affected individuals commonly show a period of apparent early normal development, followed by regression of hand and/or communication skills, and subsequent development of hand stereotypies, while gait is often abnormal in those who are learning to walk (6).

Wide variability in clinical phenotypes of patients with RTT is observed and often related to specific *MECP2* mutations (7, 8). It is currently evident that RTT is a multisystemic disease (9) with associated comorbidities often being linked to genotype-phenotype correlations (10).

In particular, sleep problems in RTT have been largely investigated using questionnaires (11–13) and performing polysomnography (14–19), electroencephalogram spectral analysis (20), or actigraphy (3). Breathing abnormalities are considered as a hallmark feature in RTT (21, 22).

Although breathing abnormalities in RTT have been reported to disappear during sleep, there are, indeed, indications of respiratory abnormalities that occur during both wakefulness and sleep (14, 23–27). Awake breathing dysfunction follows an age-dependent pattern, it occurs in vast majority of patients with RTT, and it is related to function, quality of life, and risk for cardiac dysrhythmia (28).

Earlier findings indicate that breathing disturbances in RTT can be linked to oxidative stress (OS) (29–32), abnormal erythrocyte shapes (33), pulmonary gas exchange abnormalities (32), and a pulmonary radiological picture partially mimicking that of respiratory bronchiolitis-associated interstitial lung disease (RB-ILD) (34). However, to date, no evidence exists regarding a potential differential impact of breath holding on circulating pro-oxidant molecules and acid-base balance/blood gas analysis during the sleep-wakefulness cycle in girls with RTT.

The aim of this study was to assess the clinical relevance of apneas during the sleep-wakefulness cycle in a population with RTT and the possible impact of apneas on circulating oxidative stress markers.

## Materials and Methods

### Participants

A total of (*n* = 66) female patients with a clinical diagnosis of typical RTT and proven MECP2 gene mutation were recruited (mean age: 12.6 ± 7.6 years; range: 2–32 years). RTT diagnosis and inclusion/exclusion criteria were based on the revised nomenclature consensus (6). To evaluate the clinical impact on illness severity, the Rett Clinical Severity Score (RCSS) was assessed (35). Corresponding *z*-scores for head circumference and body mass index were calculated on the basis of validated RTT-specific growth charts (36). Pain sensitivity was rated as either delayed or reduced response in 53 (81%) of the 66 of examined females with RTT. To take into account the possible influence of presence of scoliosis and epilepsy on respiratory dysfunction, data regarding coronal Cobb angle, presence of epilepsy, seizure frequency, and antiepileptic drug (AED) therapy were recorded. Scoliosis severity was rated as mild (Cobb angle 10–20^°^), moderate (Cobb angle < 40^°^), or severe (≥ 40^°^) according to Killian et al. (37). A routine clinical ENT evaluation was performed, and tonsillar size grading was categorized according to Brodsky (38). Clinical stage distribution was stage II (*n* = 10), stage III (*n* = 38), and stage IV (*n* = 18). All the patients were admitted to the Rett Syndrome National Reference Center of the University Hospital of Azienda Ospedaliera Universitaria Senese (Siena, Italy). Given the specific aim of the study, subjects with clinically evident inflammatory conditions, either acute or chronic, and individuals on anti-inflammatory drugs or undergoing supplementation with known antioxidants were excluded. At the time of examination, all the patients were neither on ventilatory assistance nor oxygen supplementation.

All the examined subjects were on a typical Mediterranean diet. The study was approved by the Ethical Committee of Siena University Hospital (Azienda Ospedaliera Universitaria Senese, Siena, Italy). Informed consent was provided by the parents/legal guardians.

### Clinical Severity

Clinical severity was assessed by Rett Clinical Severity Score (RCSS) (35), a validated RTT-specific scale designed to assess the severity of key symptoms and consists of 13 items providing a rating of core symptoms of RTT on a Likert scale of either 0–4 or 0–5 with a maximum total score of 58. RCSS was rated by two experienced clinicians (JH and CDF). To reduce interobserver variability, the average score of the two physicians was used for data analysis.

### Cardiorespiratory Monitoring

In order to analyze the occurrence of apneas and hypopneas, breathing monitoring was carried out on the patients with RTT during wakefulness and sleep state using a portable polygraphic screening device for a mean recording time of 13 ± 0.5 h for each state (SOMNOwatchTM plus; SOMNOmedics, Randersacker, Germany; importer for Italy Linde Medicalesrl). The monitoring included nasal airflow, arterial oxygen saturation by pulse oximetry, and respiratory efforts by abdominal and thoracic bands. Breathing patterns were analyzed for presence of apneas and hypopneas according to standardized definitions by the American Academy of Sleep Medicine (39) and the American Academy of Pediatrics (40).

Apneas were defined as > 90% airflow decrease for 10 s, and hypopneas were defined as > 50% airflow reduction for ~10 s associated with decrease of 3% in oxygen saturation (40). Apneas were categorized as obstructive (i.e., cessation of airflow for 10 s with persistent respiratory effort), central (i.e., cessation of airflow for 10 s with no respiratory effort), or mixed (apneas that begin as central but end up as obstructive). Apnea-hypopnea index (AHI) was the expression of the number of obstructive and central apneas and hypopneas per hour of sleep and calculated by dividing the total number of events by total sleep time. An AHI > 15 was considered clinically significant, and all records were reviewed and interpreted by an expert pneumologist (MR).

### Blood Gas Analysis (BGA) and Acid-Base Balance

Arterial blood for gas analyses was obtained from either the humeral or the radial artery prior to cardiorespiratory recording. Partial arterial pressure of oxygen (PaO_2_) and carbon dioxide (PaCO_2_), and pH were determined by a commercially available blood gas analyzer (ABL520 radiometer; Copenhagen, Denmark). In order to account for low PaCO_2_ values often observed in patients with RTT, standard PaO_2_ was calculated according to the formula by Sorbini et al. (41) [i.e., PaO_2_ = 1.66 × PaCO_2_ + PaCO_2_ – 66.4].

Patterns of BGA were categorized by a pneumologist (MR) with longstanding expertise on BGA interpretation as follows: normal acid-base balance, acute respiratory alkalosis, compensated respiratory acidosis, fully compensated respiratory alkalosis, partially compensated respiratory alkalosis, compensated respiratory alkalosis and metabolic acidosis, or mixed respiratory alkalosis/metabolic acidosis.

### Blood Cell Count

All routine clinical analytes were assayed on a Sysmex XT-2100 system (42). Mean corpuscular volume (MCV), mean cell hemoglobin (MCH), mean cell hemoglobin concentration (MCHC), and red cell distribution width (RDW) were measured with an electric resistance detecting method (impedance technology) and by hydrodynamic focusing. Hemoglobin (Hb) was measured photocolorimetrically using a cyanide-free method.

### Oxidative Stress (OS) Markers

#### Blood Sampling

Blood was collected in heparinized tubes and all procedure were carried out within 2 h after sample collection. Blood samples were centrifuged at 2,400 × *g* for 15 min at 4^°^C; platelet-poor plasma was saved, and buffy coats were removed by aspiration. RBCs were washed twice with a physiologic solution (150 mM NaCl). An aliquot of packed erythrocytes was resuspended in a Ringer solution (125 mM NaCl, 5 mM KCl, 1 mM MgSO_4_, and 32 mM N-2 hydroxyethylpiperazine-N2-ethanesulfonic acid, HEPES, 5 mM glucose, and 1 mM CaCl2), pH 7.4 as a 50% (vol/vol) suspension for the determination of intra-erythrocyte NPBI. Plasma was used for the NPBI assay and lipid peroxidation markers (F_2_-isoprostanes).

#### Intra-Erythrocyte and Plasma Non-protein-bound Iron (IE- and P-NPBI)

Non-protein-bound iron (NPBI) is considered not only an OS marker but also a pro-oxidant factor. In particular, IE-NPBI is a critical marker of hypoxia. IE-NPBI (nmol/ml erythrocyte suspension) was determined as a desferrioxamine- (DFO) iron complex (ferrioxamine), as previously reported (30). P-NPBI (nmol/ml) was determined as the above reported for IE-NPBI (30).

#### Plasma F_2_-Isoprostanes (F_2_-IsoPs)

A series of prostaglandin F_2_-like compounds (F_2_-IsoPs) generated by non-enzymatic oxidation of membrane phospholipid-bound arachidonic acid are considered specific and reliable markers for evaluation of *in vivo* OS conditions. A gas chromatography/negative ion chemical ionization tandem mass spectrometry (GC/NICI-MS/MS) analysis with prior derivatization steps were carried out. To quantify F_2_-IsoP amounts, the measured ions of 15-F_2t_-IsoPs (one of the most represented isomers of F_2_-IsoPs and also known as 8-epi-PGF2α) and internal standard PGF2α-d4 were determined [product ions at *m/z* 299 and *m/z* 303 whose precursors ions were *m/z* 569 and *m/z* 573, respectively (43)].

### Analysis of Statistical Data

All variables were tested for normal distribution (D'Agostino-Pearson test), and data were presented as means ± standard deviation or medians and interquartile range for continuous normally distributed and non-Gaussian variables, respectively. Differences between RTT and control groups were evaluated by independent-sample *t*-test (continuous normally distributed data), Mann-Whitney rank sum test (continuous non-normally distributed data), chi-square statistics (categorical variables with minimum number of cases per cell ≥ 5), or Fisher's test (categorical variables with minimum number of cases per cell 0.5 was accepted to indicate good discrimination). Relationships between variables in univariate analysis were tested by linear regression analysis or Spearman's rank correlation. Analysis of variance was performed by one-way ANOVA or Kruskal-Wallis test, as appropriate. To test independent predictor variables for a dependent variable, stepwise multiple regression analysis models (significance entry criterion *p* < 0.05, with removal criterion *p* > 0.1) were tested considering normal distribution of residuals by Kolmogorov-Smirnov test. The MedCalc version 20.013 statistical software package (MedCalc Software Ltd., Ostend, Belgium; https://www.medcalc.org; 2021) was used for data analysis, and a two-tailed *p* < 0.05 was considered to indicate statistical significance.

## Results

### Baseline Data

#### Demographics, Biometrics, and Clinical Features

Demographics, biometrics, and clinical features of the examined patients with RTT are shown in Table 1. Mean patient's age was 12.5 ± 7.4 years (pediatric patients, *n* = 50, 75.8% vs. adult patients, *n* = 16, 24.2%; chi-square 17.515, DF = 1, contingency coefficient 0.458, *p* < 0.0001).

**Table 1:** Demographic and clinical characteristics of the examined patients with Rett syndrome (RTT) (*n* = 66).

**Variables**	**Mean (range), *N* (%)**	***P*** **-value**
Age (years)	12.5 ± 7.4 (2–32)	
Head circumference (cm)	50.3 ± 2.2 (45.5–55)	
Head circumference (RTT *z*-scores^a^)	0.20 ± 0.72 (−1.28–2.05)	
BMI (RTT *z*-scores for age^a^)	0.01 [−0.81–0.91] (−1.55–1.88)	
**Disease stage**		
Stage I	0 (0.0)	**0.0001**
Stage II	10 (15.1)	
Stage III	38 (57.6)	
Stage IV	18 (27.3)	
***MECP2*** **mutation category**		
Early truncating	28 (42.4)	**0.0003**
Gene deletion	4 (6.1)	
Late truncating	14 (21.2)	
Missense	20 (30.3)	
**Scoliosis^b^**	40 (60.6)	0.085
Mild (Cobb angle 10^°^–19^°^)	12 (30.0)	
Moderate (Cobb angle 20^°^–39^°^)	14 (35.0)	0.905
Severe (Cobb angle ≥ 40^°^)	14 (35.0)	
**Epilepsy**	50 (75.8)	**<0.0001**
**Seizure frequency**		
< Monthly	14 (28)	
Weekly to monthly	6 (12)	0.1935
Weekly	14 (28)	
Daily to weekly	16 (32)	
**AED therapy**	48 (72.7)	**0.0002**
Monotherapy	24 (50)	
Multitherapy	24 (50)	
**RCSS**	24.0 ± 7.6 (12–44)	

*Qualitative data were described using number and percentage. Quantitative data were expressed as mean ± SD or median [inter-quartile range]*.

a *Calculated z-scores for age are referred to a validated Rett syndrome-specific growth chart (36)*.

b *Classified according to the Cobb angle value (37)*.

*BMI, body mass index; RCSS, Rett Clinical Severity Score; AED, antiepileptic drugs*.

*Bold characters indicate statistically significant differences*.

After RTT curve-specific BMI *z*-score categorization into low (< −1.881, i.e., <3rd percentile), normal (−1.881 < *z*-score <1.881), and high (>1.881, i.e., > 97th percentile) range groups, a significant difference was observed with 6.1% in the high-range group (*n* = 4) and 93.9% in the normal range (*n* = 62) without patients in the low-range group. (chi-square 50.97, DF = 1, contingency coefficient 0.66, *p* > 0.0001) (data not shown).

No difference was observed for the presence of scoliosis (40 of the 66 patients with RTT have scoliosis vs. 26 who have no scoliosis, chi-square 2.97, DF = 1, contingency coefficient 0.208, *p* = 0.0848) or for its degree [mild (*n* = 12, 30%), moderate (*n* = 14, 35%), and severe (*n* = 14, 35%) (chi-square 0.2, DF = 2, contingency coefficient 0.071, *p* = 0.071)].

A total of 9 out of the 66 patients with RTT (22%; mean age 10.1 ± 3.4 yrs) exhibited tonsillar hypertrophy on clinical examination (*n* = 4 tonsil size grading scale 1+, i.e., tonsils occupying <25% of the lateral dimension of the oropharynx, as measured between the anterior tonsillar pillars; and *n* = 5 tonsil size grading scale 2+, i.e., tonsils occupying 26–50% of the lateral dimension of the oropharynx) (38).

Of the examined patient's population (*n* = 66), 75.8% (50/66) exhibited seizures vs. 24.2% (16/66) with no seizures. Among the patients with RTT with seizures, 28% (14/50) had seizure frequency < monthly, 12% (6/50) weekly to monthly, 28% (14/50) weekly, and 32% (16/50) daily to weekly.

A total of 48 out of the 66 patients were on AEDs (*p* = 0.002): 24/48 were on monotherapy and 24/48 were on multi-therapy. Among the AED-treated patients, 42/48 (87.5%) were on carbamazepine (CBZ), 8/48 (16.7%) on valproic acid (VPA), 10/48 (20.8%) on topiramate (TPM), 4/48 (8.3%), on levetiracetam (LEVET), 2/48 (4.2%), on clobazam (CLB), and 8/48 (16.7%) on phenobarbital (PB).

Significant differences in the distribution of clinical disease stages (significant excess of patients in stage III, i.e., 57.6%, chi-square 18.909, DF = 2, contingency coefficient 0.472, *p* = 0.0001) and *MECP2* mutation category (significant excess in early truncating mutations, i.e., 42.4%, chi-square 18.606, DF = 3, contingency coefficient 0.469, *p* = 0.0003) were observed.

Based on the *MECP2* genotype, the patients with RTT harbored the following *MECP2* pathogenic mutations: R106W (3%), T158M (12.1%), R168X (6.1%), R255X (12.1%), R270X (12.1%), R294X (6.1%), C-terminal deletions (18.2%), large deletions (6.1%), and non-hotspot mutations (24.2%).

An *MECP2* genotype-related distribution of scoliosis frequency was observed (chi-square 21.818, DF = 8, *p* = 0.0053) with the highest frequency in patients with RTT with non-hotspot-type mutations (24.2%), C-terminal deletions (18.2%), R255X (12.1%), R270X (12.1%), and T158M (12.1%). The lowest frequency of scoliosis was observed in patients with late truncating deletions (6.1%), R168X (6.1%), R294X (6.1%), and R106W (3%).

#### Cardiorespiratory Monitoring and Arterial Blood Gas Analysis

Baseline cardiorespiratory monitoring data are shown in Table 2. The patients with RTT displayed an excess in obstructive apneas (80.3%) compared to the central and mixed ones (chi-square 66.091, DF = 2, contingency coefficient 0.707, *p* < 0.0001). Significant prevalence of AHI values > 15 was observed in 69.7% of the whole population with RTT (chi-square 10.242, DF = 1, contingency coefficient 0.367, *p* = 0.0014).

**Table 2:** Baseline cardiorespiratory monitoring of the examined patients with RTT (*n* = 66).

**Variables**	**Mean (range), *N* (%)**
Apneas^**^	
Obstructive	53 (80.3)
Central	4 (6.1)
Mixed	9 (13.6)
AHI > 15^*^	46 (69.7)
Total breath holding episodes	59 [13–178]
Total desaturation episodes	11 [2.5–36.5]
Average SpO_2_ values (%)	97 [95–98]
Nadir SpO_2_ values (%)	68.7 ± 23.7

*Qualitative data are described using number and percentage. Normally quantitative data are expressed as mean ± SD or median [inter-quartile range]. Apneas were defined as a > 90% airflow decrease for ≥ 10 s (38). Obstructive apneas refer to recorded events with cessation of airflow for ≥ 10 s associated with persistent respiratory effort; central apneas refer to events characterized by cessation of airflow for ≥ 10 s without associated respiratory effort; mixed apneas refer to respiratory events that begin as central apneas and end up as obstructive apneas*.

* *p = 0.0014*.

** *p < 0.0001*.

*AHI, apnea-hypopnea index; SpO_2_, peripheral oxygen saturation; nadir SpO_2_, lowest values of SpO_2_ during the cardiorespiratory recordings*.

No significant difference was observed in total breath holding episodes as a function of disease stage (Kruskal-Wallis test *p* = 0.1409). Total breath holding episodes are statistically different as a function of *MECP2* genotype distribution (Kruskal-Wallis test *p* = 0.0329). Highest number of total breath holding episodes were recorded in patients harboring both R270X mutations [134 (inter-quartile range, IQR, 65–274)] and C-terminal deletions [13.5 (IQR 1–194)], while lowest breath holding number was recorded in patients with other non-hotspot mutation types [30.5 (IQR 21–44)] and T158M [32.5 (IQR 10.5–108.5)] (Supplementary Figure 1). Total breath holding episodes were positively related to disease severity (*r* = 0.307, *p* = 0.0121) (Supplementary Figure 2).

Total desaturation episodes were significantly different as a function of disease stage (stage II median 11.5, stage III 7.5, and stage IV 40; Kruskal-Wallis *p* = 0.00001) with highest values in stages II and IV (stages II and III vs. stage IV, Conover test *p* <0.05) (Supplementary Figure 1). No significant differences were observed in *MECP2* genotype distribution for total desaturation episodes (Kruskal-Wallis test *p* = 0.6219). Total desaturation episodes were positively correlated to disease severity (*r* = 0.6193, *p* < 0.0001) (Supplementary Figure 2).

No significant difference was observed in average SpO_2_ values as a function of disease stage (Kruskal-Wallis test *p* = 0.581). An *MECP2* genotype-related distribution of average SpO_2_ was observed (Kruskal-Wallis test *p* = 0.0423), with the highest values in patients with R168X mutations [99 (IQR 99–99)], R294X mutations [97.5 (IQR 97–98)], and other non-hotspot-type mutations [97 (IQR 96–98.5)]. In contrast, the lowest values of average SpO_2_ were observed in patients with R255X mutations [95.5 (IQR 94–96.5)], large deletions [94.5 (IQR 93–97)], and R106W mutations [94 (IQR up to 94)] (Supplementary Figure 1). Average SpO_2_ values were negatively correlated to age (*r* = −0.2645, *p* = 0.032) (Supplementary Figure 2), while no correlation was observed between average SpO_2_ values and clinical severity (*r* = −0.141, *p* = 0.259).

No significant difference was observed in nadir SpO_2_ values as a function of disease stage (Kruskal-Wallis test *p* = 0.6274). Nadir SpO_2_ (i.e., lowest values of SpO_2_ reached during cardiorespiratory recordings) values were unevenly distributed according to *MECP2* genotype (Kruskal-Wallis test *p* = 0.0062). Nadir SpO_2_ values close to physiological range were recorded in patients with R106W mutations [89 (IQR up to 89)], other non-hotspot-type mutations [87 (IQR 86–94)], and C-terminal deletions [85.5 (IQR 78–90)], whereas values farther from the physiological range were observed in patients harboring R255X mutations [58.5 (IQR 33–84.5)], large deletions [53 (IQR 48–63)], and R270X mutations [45 (IQR 31.5–72)] (Supplementary Figure 1). A non-significant correlation was observed between nadir SpO_2_ values and clinical severity (*r* = −0.175, *p* = 0.16).

#### Arterial Blood Gas Analysis

**Figure 1:**
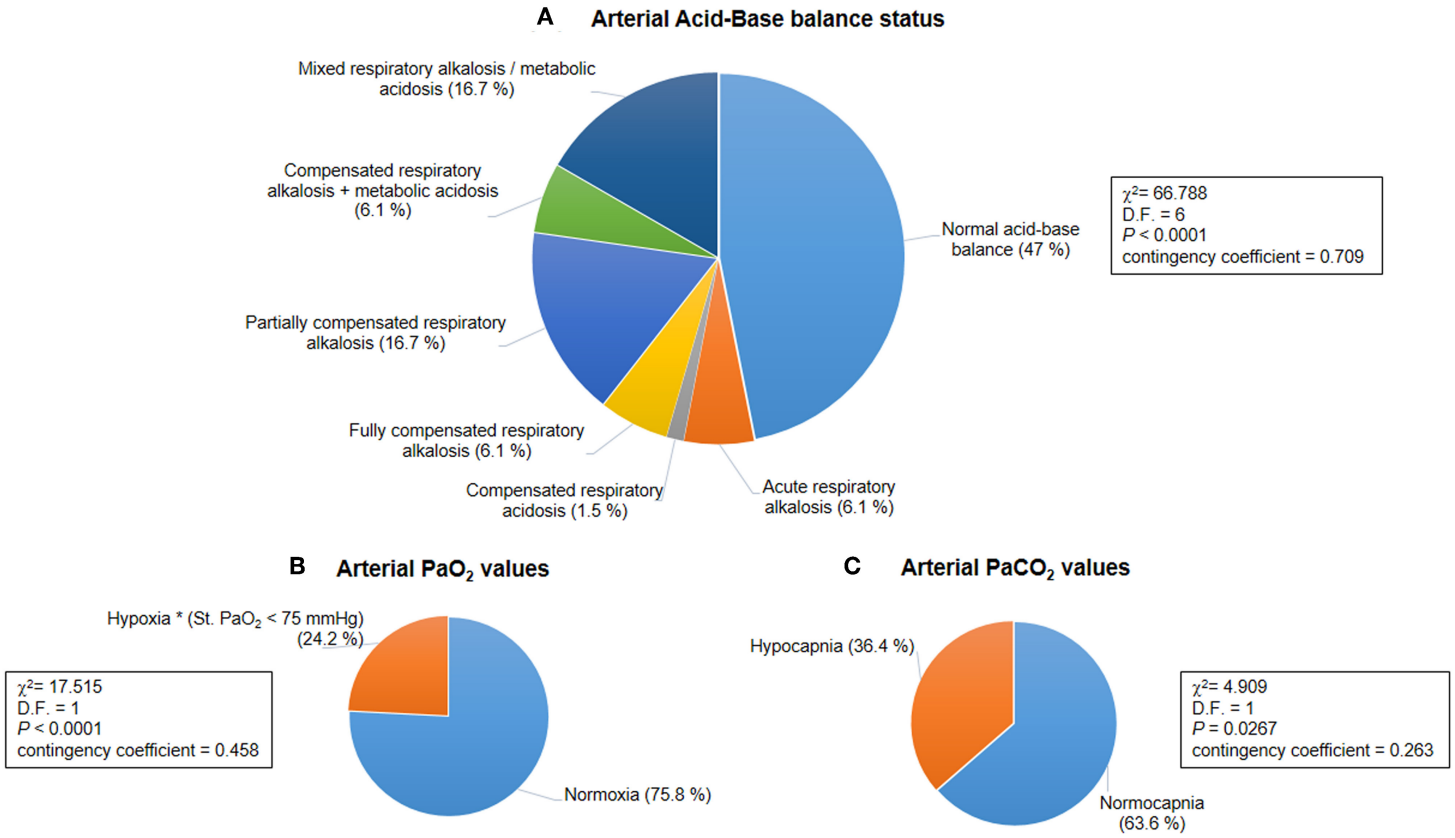
Baseline acid-base balance and gas analysis status for the whole population with Rett syndrome (RTT) (*n* = 66). **(A)** Distribution of patterns of the arterial acid-base balance status was significantly different (*p* < 0.0001). **(B)** A hypoxic condition (i.e., arterial blood Std. PaO_2_ < 75 mmHg) was observed in 24.2% of the whole population with RTT (*p* < 0.0001). **(C)** Hypocapnia (i.e., arterial blood PaCO_2_ < 35 mmHg) was present in 36.4% of all the examined patients with RTT (*p* = 0.0267). PaO_2_, partial arterial pressure of oxygen; PaCO_2_, partial arterial pressure of carbon dioxide.

Results of the blood gas analyses are shown in Table 3. Baseline hypoxia (i.e., standard PaO_2_ < 75 mmHg) and hypocapnia (i.e., blood PaCO_2_ < 35 mmHg) were present in *n* = 16 and *n* = 24 of the patients with RTT, respectively (standard PaO_2_: chi-square 17.515, DF = 1, contingency coefficient 0.458, *p* < 0.0001; PaCO_2_: chi-square 4.909, DF = 1, contingency coefficient 0.263, *p* = 0.0267) (Figure 1). Interestingly, no significant relationships were observed between average SpO_2_ and blood PaO_2_ or standard PaO_2_ (*r* = −0.069, *p* = 0.5831 and *r* = −0.038, *p* = 0.7641, respectively). No significant difference was detected (*p* =0.172) concerning normalized respiratory rate (*z*-scores for age) as a function of hypocapnia. Interestingly, blood standard PaO_2_ values were significantly decreased in RTT patients with hypocapnia compared to patients with normocapnia (Mann-Whitney test, *p* = 0.0001) (Supplementary Figure 3).

**Table 3:** Baseline respiratory and arterial blood gas analysis of the examined patients with RTT (*n* = 66).

**Variables**	**Mean (range)**
Respiratory rate (breaths/min)	22.1 ± 4.4 (16–32.5)
Normalized respiratory rate (*z*-scores for age)^a^	0.29 ± 1.08 (−1.80–3.50)
pH	7.421 [7.400–7.450] (7.360–7.580)
PaCO_2_ (mmHg)	37.9 [31.2–41.8] (17.7–44.4)
PaO_2_ (mmHg)	91.5 [84.8–98.2] (46.7–124.6)
Std. PaO_2_ (mmHg)^*^	85.9 [79.0–90.1] (29.9–98.5)
${\text{HCO}}_{3}^{-}$ (mmol/L)	18.0 [17.0–21.0] (14.0–24.0)

*Data are expressed as mean ± standard deviation or median [inter-quartile range]*.

a *Calculated z-scores for age are referred to a validated Rett syndrome-specific growth chart (36)*.

* *Values calculated according to the formula by Sorbini et al. (41) accounting for hypocapnia: standard PaO_2_ = 1.66 × PaCO_2_ + PaO_2_ – 66.4*.

*PaCO_2_, partial arterial pressure of carbon dioxide; PaO_2_, partial arterial pressure of oxygen; Std. PaO_2_, standard PaO_2_, ${\text{HCO}}_{3}^{-}$, bicarbonate*.

Significant positive correlations were observed for standard PaO_2_, blood ${\text{HCO}}_{3}^{-}$ and disease severity (*r* = 0.247, *p* = 0.045 and *r* = 0.261, *p* = 0.0344, respectively) (Supplementary Figure 2).

Based on BGA pattern interpretation (Figure 1), *n* = 31 of the patients with RTT presented normal acid-base balance, whereas *n* = 35 showed altered BGA patterns. In particular, the following patterns were observed: acute respiratory alkalosis (*n* = 4), compensated respiratory acidosis (*n* = 1), fully compensated respiratory alkalosis (*n* = 4), partially compensated alkalosis (*n* = 11), compensated respiratory alkalosis and metabolic acidosis (*n* = 4), and mixed respiratory alkalosis/metabolic acidosis (*n* = 11). The distribution of the patterns was statistically significant (chi-square 66.788, DF = 6, contingency coefficient 0.709, *p* < 0.0001).

**Figure 2:**
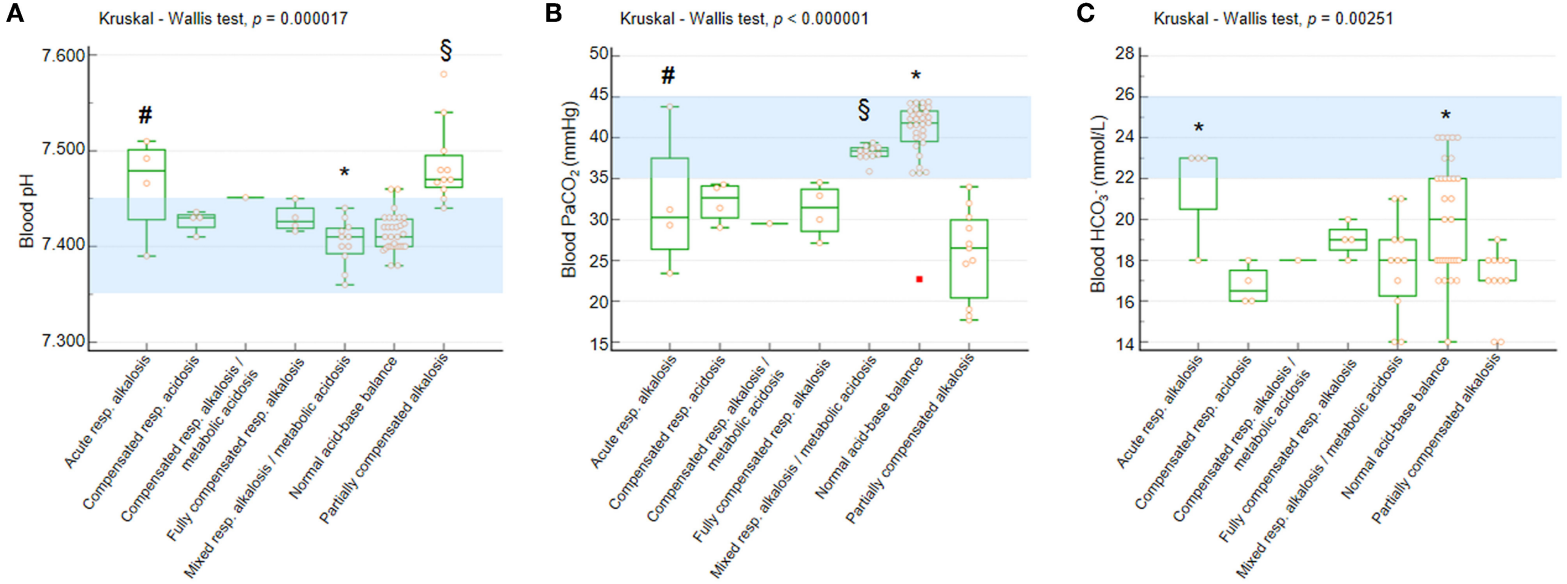
Blood pH, PaCO_2_, and ${\text{HCO}}_{3}^{-}$ in the whole population with RTT as a function of acid-base balance patterns. **(A)** Blood pH. *Post-hoc* analysis (Conover test): *p < 0.05 vs. all the other categories with the single exception of normal acid-base balance. ^§^vs. compensated respiratory alkalosis + metabolic acidosis, fully compensated respiratory alkalosis, mixed respiratory alkalosis/metabolic acidosis, and normal acid-base balance. ^#^vs. normal acid-base balance. **(B)** Blood PaCO_2_. *Post-hoc* analysis (Conover test):*p < 0.05 vs. all the other categories. ^§^vs. compensated respiratory alkalosis + metabolic acidosis, fully compensated respiratory alkalosis, and partially compensated respiratory alkalosis. ^#^vs. partially compensated respiratory alkalosis and normal acid-base balance. **(C)** Blood ${\text{HCO}}_{3}^{-}$. *Post-hoc* analysis (Conover test): *p < 0.05 vs. compensated respiratory alkalosis + metabolic acidosis, mixed respiratory alkalosis/metabolic acidosis, and partially compensated respiratory alkalosis. ^§^vs. compensated respiratory alkalosis + metabolic acidosis and partially compensated respiratory alkalosis. PaCO_2_, partial arterial pressure of carbon dioxide; ${\text{HCO}}_{3}^{-}$, arterial bicarbonate. Data are shown as box and whisker plots. Bars represent medians and inter-quartile ranges. Red rectangle indicates outlier data point. Normal range values (consensus definitions) are highlighted in light blue.

Significant differences were observed for blood pH, PaCO_2_, and ${\text{HCO}}_{3}^{-}$ values as a function of BGA pattern (Kruskal-Wallis test *p* = 0.000017, *p* < 0.000001, and *p* = 0.002515, respectively) (Figure 2).

All the patients showed blood pH within the reference range with the exception of girls with acute respiratory alkalosis and partially compensated alkalosis (Figure 2A). Only patients with normal acid-base balance and mixed respiratory alkalosis/metabolic acidosis showed blood PaCO_2_ levels within the normal range, whereas the other categories showed median values below the lower normal limit (Figure 2B).

The patients showed reduced blood ${\text{HCO}}_{3}^{-}$ levels with the exception of those with acute respiratory alkalosis (Figure 2C).

According to prevalence of breath holding, the RTT population was subsequently categorized into two main apnea subgroups i.e., wakefulness apnea (WA) or sleep apnea (SA).

Distribution of normalized respiratory rate (i.e., *z*-scores) as a function of acid-base balance status is shown (Supplementary Figure 4A), as well as an exploratory study on the relationships between normalized respiratory rate (*z*-scores) and arterial blood gas parameters in the whole population with RTT (Supplementary Figures 4B–E). Although no significant differences were detectable, higher normalized respiratory rate was observed in patients with acute respiratory alkalosis (*p* = 0.052).

#### Red Blood Cell Parameters

Baseline hematological variables are shown in Table 4. Overall, a condition of apparently asymptomatic anemia was found to be present in 19 (28.8%) of the whole population (chi-square 42.818, DF = 2, contingency coefficient 0.627, *p* < 0.0001). The degree of anemia was found to be mild in 8 patients (12.1% of the whole population and 42.1% of the anemic patients) and moderate in 11 patients (16.7% of the whole population and 57.9% of the anemic patients with RTT). A total of 13 girls of pediatric age and 6 adult patients showed Hb levels below the WHO cut-off diagnostic criteria for anemia laboratory diagnosis (44) (chi-square 1.046, DF = 2, contingency coefficient 0.593, *p* = 0.528).

**Table 4:** Baseline red blood cell count variables of patients with RTT (*n* = 66).

**Variables**	**Mean (range)**
Hb (g/dL)	12.8 [11.1–13.2] (9.4–14.5)
MCV (fL)	84.7 [81.9–90.1] (61.7–96.2)
MCH (pg/cell)	27.7 [26.6–30.0] (20.0–31.7)
MCHC (g/dL)	32.8 [31.7–33.2] (29.7–34.4)
RDW (%)	13.8 [13.1–14.9] (11.9–22.0)

*Data are expressed as median [inter-quartile range]*.

*Hb, hemoglobin; MCV, mean corpuscular volume; MCH, mean cell hemoglobin; MCHC, mean cell hemoglobin concentration; RDW, red cell distribution width*.

No significant differences were observed in the frequency of asymptomatic anemia (*n* = 19) vs. non-anemic patients (*n* = 47) as a function of RTT curve-specific BMI *z*-score category (anemic patients with RTT: high BMI range group *n* = 2 and normal BMI range group *n* = 17) (chi-square 0.92, DF = 1, contingency coefficient 0.117, *p* = 0.337). As it concerns the degree of anemia, no significant differences were observed (mild anemia: *n* = 2 high BMI range group vs. *n* = 6 normal BMI range group; moderate anemia: all the patients (*n* = 11) were within the normal BMI range group) (chi-square 2.912, DF = 1, contingency coefficient 0.365, *p* = 0.088).

The distribution of anemic vs. non-anemic patients as a function of scoliosis was significantly asymmetric; indeed, scoliosis was observed in 16/19 (84.2%) anemic patients vs. 24/47 (51.1%) non-anemic patients (chi-square 6.132, DF = 1, contingency coefficient 0.292, *p* = 0.0133). When the degree of anemia is considered, no significant difference was observed between the groups (mild anemia group: 6/8 patients presented scoliosis vs. moderate anemia group: 10/11) (chi-square 0.835, DF = 1, contingency coefficient 0.361, *p* = 0.205).

### Wakefulness Apnea (WA) vs. Sleep Apneas (SA)

#### Demographics, Biometrics, and Clinical Features

Demographics, biometrics, and clinical features of the WA (*n* = 17) and SA (*n* = 49) subgroups are shown in Table 5. Mean age of the WA subgroup was 14.7 ± 9.2 years (pediatric patients, *n* = 11, 64.7%, vs. adult patients, *n* = 6, 35.3%), whereas the mean age of the SA subgroup was 11.8 ± 6.6 years (pediatric patients, *n* = 39, 79.6%, vs. adult patients, *n* = 10, 20.4%). No significant difference was observed in the WA and SA subgroups (chi-square 1.5, DF = 1, contingency coefficient 0.149, *p* = 0.2207).

**Table 5:** Demographic and clinical characteristics of the examined population with RTT as a function of wakefulness (*n* = 17) vs. sleep apneas (*n* = 49).

**Variables**	**Apneas**		***P*** **-value**
	**Wakefulness**	**Sleep**	
	**Mean (range), *N* (%)**	**Mean (range), *N* (%)**	
Age (years)	14.7 ± 9.2 (6.0–32)	11.8 ± 6.6 (2.0–24)	0.165
Head circumference (cm)	50.5 ± 1.9 (47.0–55.0)	50.2 ± 2.2 (45.5–55.0)	0.593
Head circumference (RTT *z*-scores^a^)	0.31 ± 0.75 (−1.89–2.05)	0.16 ± 0.72 (−1.28–2.05)	0.468
BMI (RTT *z*-scores for age^a^)	0.38 [−0.92–1.06] (−1.40–1.64)	−0.02 [−0.79–0.91] (−1.55–1.88)	0.786
**Disease stage**			
Stage I	0 (0.0)	0 (0.0)	
Stage II	2 (11.8)	8 (16.3)	0.781
Stage III	11 (64.7)	27 (55.1)	
Stage IV	4 (23.5)	14 (28.6)	
***MECP2*** **mutation category**			
Early truncating	6 (35.3)	19 (41.3)	
Gene deletion	0 (0.0)	4 (8.7)	0.283
Late truncating	6 (35.3)	8 (17.4)	
Missense	5 (29.4)	15 (32.6)	
**Scoliosis^b^**	9 (52.9)	31 (63.3)	0.456
Mild (Cobb angle 10^°^–19^°^)	1 (11.1)	11 (35.5)	
Moderate (Cobb angle 20^°^–39^°^)	4 (44.4)	10 (32.3)	0.373
Severe (Cobb angle ≥ 40^°^)	4 (44.4)	10 (32.3)	
**Epilepsy**	11 (64.7)	39 (79.6)	0.221
**Seizure frequency**			
< Monthly	2 (18.2)	12 (30.8)	
Weekly to monthly	2 (18.2)	4 (10.3)	0.346
Weekly	5 (45.5)	9 (23.1)	
Daily to weekly	2 (18.2)	14 (35.9)	
**AED therapy**			
Monotherapy	5 (45.4)	19 (51.4)	0.734
Multitherapy	6 (54.5)	18 (48.6)	
**RCSS**	22.9 ± 5.6 (12–32)	24.4 ± 8.2 (14–44)	0.479

*Qualitative data are described using number and percentage. Normally quantitative data are expressed as mean ± SD*.

a *Calculated z-scores for age are referred to a validated Rett syndrome specific growth chart (36)*.

b *Classified according to the Cobb angle value (37)*.

*BMI, body mass index; RCSS, Rett Clinical Severity Score; AED, antiepileptic drugs*.

After RTT curve-specific BMI *z*-score categorization (low-, normal-, and high-range groups), no significant difference was observed between the patients with WA and SA [WA: normal, 100% (*n* = 17); SA: high, 8.2 (*n* = 4), and 91.8% (*n* = 45) normal; chi-square 1.455, DF = 1, contingency coefficient 0.147, *p* = 0.2277]. There was no significant difference in the distribution of tonsillar hypertrophy between the WA and SA subgroups (WA: *n* = 1 and SA: *n* = 8, Fisher test *p* = 0.4275).

Based on *MECP2* genotype classification, no statistical difference was observed between the WA and SA subgroups (*p* = 0.0809). In particular, patients from the WA subgroup harbored the following *MECP2* pathogenic mutations: R106W (11.8%), T158M (11.8%), R168X (8.2%), R255X (11.8%), R270X (11.8%), R294X (8.2%), C-terminal deletions (35.3%), and other mutation types (17.6%). *MECP2* genotype distribution in the SA subgroup was as follows: T158M (12.2%), R255X (12.2%), R270X (12.2%), large deletions (8.2%), C-terminal deletions (12.2%), and other non-hotspot mutation types (26.5%). No significant differences in the distribution of clinical disease stages and *MECP2* mutation category were observed in the WA and SA subpopulations (*p* = 0.781 and *p* = 0.283, respectively).

#### Cardiorespiratory Monitoring

Cardiorespiratory monitoring data are shown in Table 6. In both subgroups, the patients with RTT showed an excess in obstructive apneas (94.1% in the WA subgroup vs. 75.5% in the SA subgroup) compared to the central (0% in the WA subgroup vs. 8.2% in the SA subgroup) and mixed ones (5.9% in the WA subgroup vs. 16.3% in the SA subgroup) (intergroup difference *p* = 0.2297).

**Table 6:** Cardiorespiratory monitoring of the examined population with RTT as a function of wakefulness (*n* = 17) vs. sleep apneas (*n* = 49).

**Variables**	**Apneas**		***P*** **-value**
	**Wakefulness**	**Sleep**	
	**Mean (range), *N* (%)**	**Mean (range), *N* (%)**	
**Apneas**			
Obstructive	16 (94.1)	37 (75.5)	
Central	0 (0.0)	4 (8.2)	0.2297
Mixed	1 (5.9)	8 (16.3)	
AHI > 15	7 (41.2)	39 (79.6)	**0.0032**
Total breath holding episodes	21 (12–44)	79 (55–114)	**0.0007**
Total desaturation episodes	11 (6–14)	13 (8–16)	0.5468
Average SpO_2_ values (%)	95 (94–95)	97 (97–98)	**0.0001**
Nadir SpO_2_ values (%)	88 (85.1–90)	68 (63.3–83)	**0.0004**

*Qualitative data were described using number and percentage. Normally quantitative data were expressed as median [95% CI for median]*.

*Bold characters indicate statistically significant differences (chi-square or Mann–Whitney U-test)*.

*Obstructive apneas refer to recorded events with cessation of airflow for ≥ 10 s associated with persistent respiratory effort; central apneas refer to events characterized by cessation of airflow for ≥ 10 s without associated respiratory effort; mixed apneas refer to respiratory events that begin as central apneas and end up as obstructive apneas*.

*AHI, apnea-hypopnea index; SpO_2_, peripheral oxygen saturation; nadir SpO_2_, lowest values of SpO_2_ during the cardiorespiratory monitoring*.

The SA group showed significantly increased AHI values > 15 (*p* = 0.0032), total breath holding episodes (*p* = 0.007), and average SpO_2_ (*p* = 0.0001) as well as nadir SpO_2_ (*p* = 0.0004) compared with the patients with WA. Paradigmatic examples of the distribution pattern for respiratory events in the subpopulations with WA and SA-RTT are shown in Supplementary Figure 5.

#### Blood Gas Analysis

**Figure 3:**
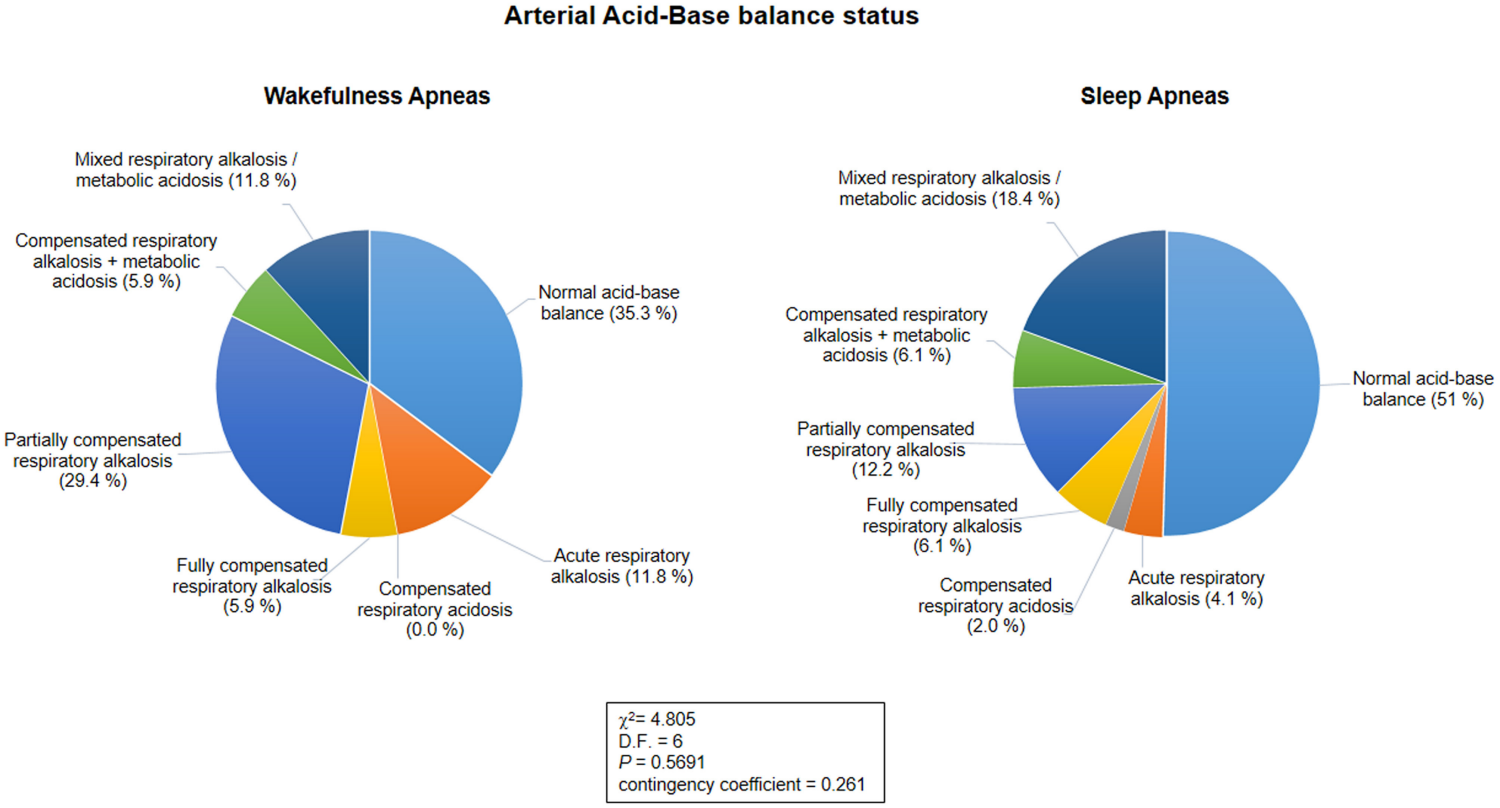
Arterial acid-base balance status as a function of the sleep-wakefulness cycle (*n* = 66). No statistical differences were observed in arterial acid-base balance patterns between the wakefulness and sleep apnea subpopulations (*p* = 0.5691).

Blood gas analysis results are shown in Table 7. No differences were observed for blood pH, PaCO_2_, and PaO_2_ between the patients with WA and those with SA (*p* ≥ 0.089). No difference was observed for frequency of hypoxia (i.e., standard PaO_2_ < 75 mmHg) (*n* = 5 in the WA subgroup and *n* = 11 in the SA subgroup; chi-square 0.328, DF = 1, contingency coefficient 0.07, *p* = 0.567). In contrast, a significant difference was observed regarding frequency of hypocapnia (*n* = 10 in the WA subgroup, *n* = 14 in the SA subgroup; chi-square 4.916, DF = 1, contingency coefficient 0.263, *p* = 0.027). Based on BGA pattern interpretation (Figure 3), no significant difference between the WA and SA subgroups was observed (*p* = 0.5691).

**Table 7:** Respiratory and arterial blood gas analysis data of the examined patients with RTT as a function of wakefulness (*n* = 17) vs. sleep apneas (*n* = 49).

**Variable**	**Apneas**		***P*** **-value**
	**Wakefulness**	**Sleep**	
	**Mean (range), *N* (%)**	**Mean (range), *N* (%)**	
Respiratory rate (breaths/min)	22.1 ± 4.8 (16–32.5)	22.1 ± 4.4 (16–32.5)	0.970
Normalized respiratory rate (*z*-scores for age)^a^	0.23 ± 0.95 (−1.0–2.0)	0.31 ± 1.12 (−1.8–3.5)	0.414
pH	7.430 [7.407–7.468] (7.380–7.581)	7.420 [7.400–7.437] (7.362–7.541)	0.129
PaCO_2_ (mmHg)	33.9 [26.9–40.6] (18.2–44.4)	38.4 [33.9–41.9] (17.7–44.3)	0.089
PaO_2_ (mmHg)	89.8 [84.9–106.6] (55.4–124.6)	92.0 [84.2–98.0] (46.7–123)	0.747
Std. PaO_2_ (mmHg)^*^	85.0 [72.4–89.4] (30.8–98.0)	86.0 [80.5–91.3] (30.0–98.5)	0.557
${\text{HCO}}_{3}^{-}$ (mmol/L)	18.0 [17.0–20.2] (14–23)	18.0 [17.7–22.0] (14–24)	0.254

*Data are expressed as mean ± standard deviation*.

a *Calculated z-scores for age are referred to a validated Rett syndrome specific growth chart (36)*.

* *Values calculated according to the formula by Sorbini et al. (41) accounting for hypocapnia: standard PaO_2_ = 1.66 × PaCO_2_ + PaO_2_ – 66.4*.

*PaCO_2_, partial arterial pressure of carbon dioxide; PaO_2_, partial arterial pressure of oxygen; Std. PaO2, standard PaO_2_; ${\text{HCO}}_{3}^{-}$, bicarbonate*.

#### Red Blood Cell Parameters

Hematological variables are shown in Table 8. No difference was observed between the WA and SA subgroups concerning Hb, MCV, and MCHC (*p* ≥ 0.083). Decreased MCH values were observed in the patients with WA compared to the patients with SA (*p* = 0.038). Although not statistically significant, the WA subgroup showed higher RDW values than the patients with SA (*p* = 0.064). Likewise, no statistically significant differences between the two subgroups in degree of anemia were detectable (WA subgroup: *n* = 4 mild, *n* = 3 moderate, vs. SA subgroup: *n* = 4 mild, *n* = 8 moderate; chi-square 0.974, DF = 1, contingency coefficient 0.221, *p* = 0.324).

**Table 8:** Red blood cell count variables in data of the examined patients with RTT as a function of wakefulness (*n* = 17) vs. sleep apneas (*n* = 49).

	**Apneas**		
**Variables**	**Wakefulness apneas**	**Sleep apneas**	***P*** **-value**
	**Mean (range), *N* (%)**	**Mean (range), *N* (%)**	
Hb (g/dL)	12.9 [11.0–13.1] (9.5–14.5)	12.8 [11.9–13.3] (9.4–14.5)	0.499
MCV (fL)	84.4 [73.4–87.9] (61.7–93.1)	84.8 [82.8–92.1] (69.2–96.2)	0.0835
MCH (pg/cell)	27.0 [22.0–28.3] (20.0–31.7)	28.4 [27.4–30.0] (20.5–31.4)	**0.038**
MCHC (g/dL)	32.5 [31.1–34.0] (30–34.4)	32.8 [32.0–33.2] (29.7–34.4)	0.724
RDW (%)	15.6 [13.2–17.5] (12.8–22.0)	13.8 [13.1–14.6] (11.9–18.9)	0.064

*Data are expressed as median [inter-quartile range]*.

*Bold characters indicate statistically significant differences (Mann–Whitney U-test)*.

*Hb, hemoglobin; MCV, mean corpuscular volume; MCH, mean cell hemoglobin; MCHC, mean cell hemoglobin concentration; RDW, red cell distribution width*.

### Circulating OS Markers

The patients with RTT showed higher levels of pro-oxidant free redox iron (i.e., NPBI) in plasma and erythrocyte suspension compared to the normal range. In particular, P-NPBI distribution was non-gaussian. Given the well-recognized risks linked to data transformation (45), an analysis of the effects of log-transformation was performed (Supplementary Figure 6), indicating correction of normality.

**Figure 4:**
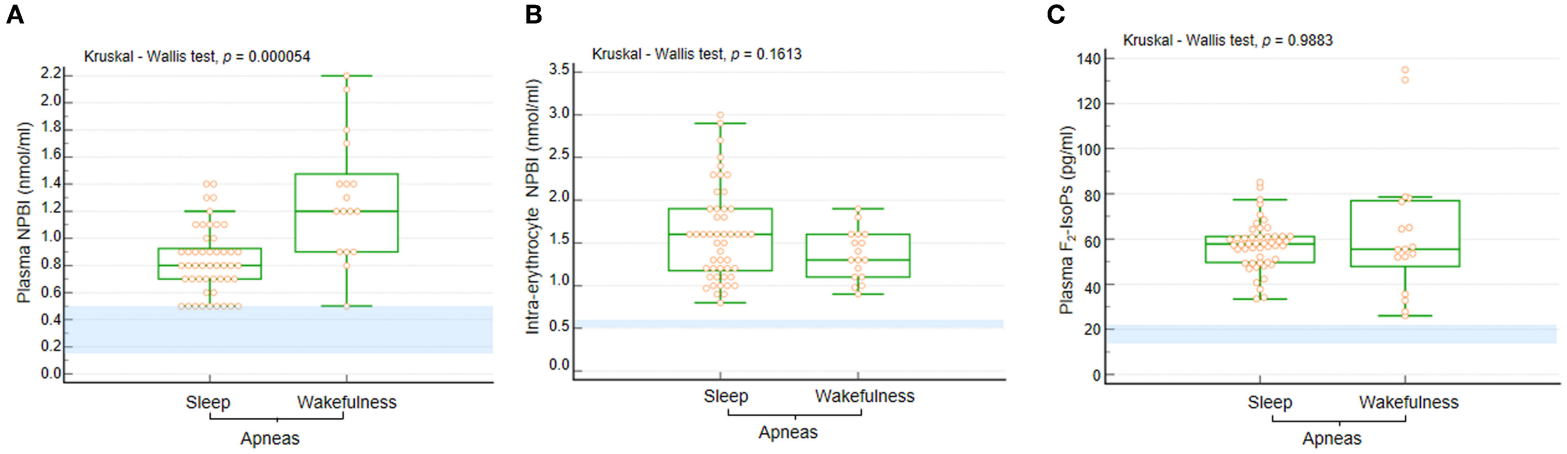
**(A)** Plasma NPBI levels were increased when apneas were predominant in wakefulness compared to those occurring in sleep. No statistically significant differences were observed for **(B)** IE-NPBI and **(C)** plasma F_2_-IsoP levels between wakefulness and sleep. Data are shown as bar graphs. Dots represent data points. Bars represent medians and intervals inter-quartile ranges. P-NPBI, plasma non-protein-bound iron; IE-NPBI, intra-erythrocyte non-protein-bound iron; F_2_-IsoPs, F_2_-isoprostanes. Normal range values are highlighted in light blue for comparison (data from an unpublished personal laboratory data set, authors: SL, CS, and LC; *n* = 45 healthy female control subjects with mean age comparable to that of the study group patients; individual data not shown).

In the whole RTT population, plasma NPBI, and intra-erythrocyte NPBI levels were 0.9 [IQR 0.7–1.2] (0.5–2.2 nmol/ml) and 1.53 ± 0.55 (0.8–3 nmol/ml) erythrocyte suspension, respectively. Likewise, plasma F_2_-IsoPs levels were found to be elevated: 57.4 [IQR 49.7–64.3] (26–135 pg/ml). In Figure 4, data ranges from an unpublished personal laboratory data set (SL, CS, and LC) of *n* = 45 healthy female control subjects with mean age comparable to that of the study group patients are used for comparison (data not shown).

Levels of P-NPBI were significantly different according to disease stage (Kruskal-Wallis test, *p* = 0.049), with highest values observed in disease stages III (median 0.9 [IQR 0.7–1.2]) and IV patients (median 0.9 [IQR 0.8–1.2]) (Supplementary Figure 7). In contrast, no significant difference was observed in P-NPBI levels in the whole RTT population as a function of presence of anemia (non-anemic, median 0.9 [IQR 0.7–1.2]) vs. anemic, median 0.9 [IQR 0.72–1.1], Mann-Whitney *U*-test *p* = 0.8249).

**Figure 5:**
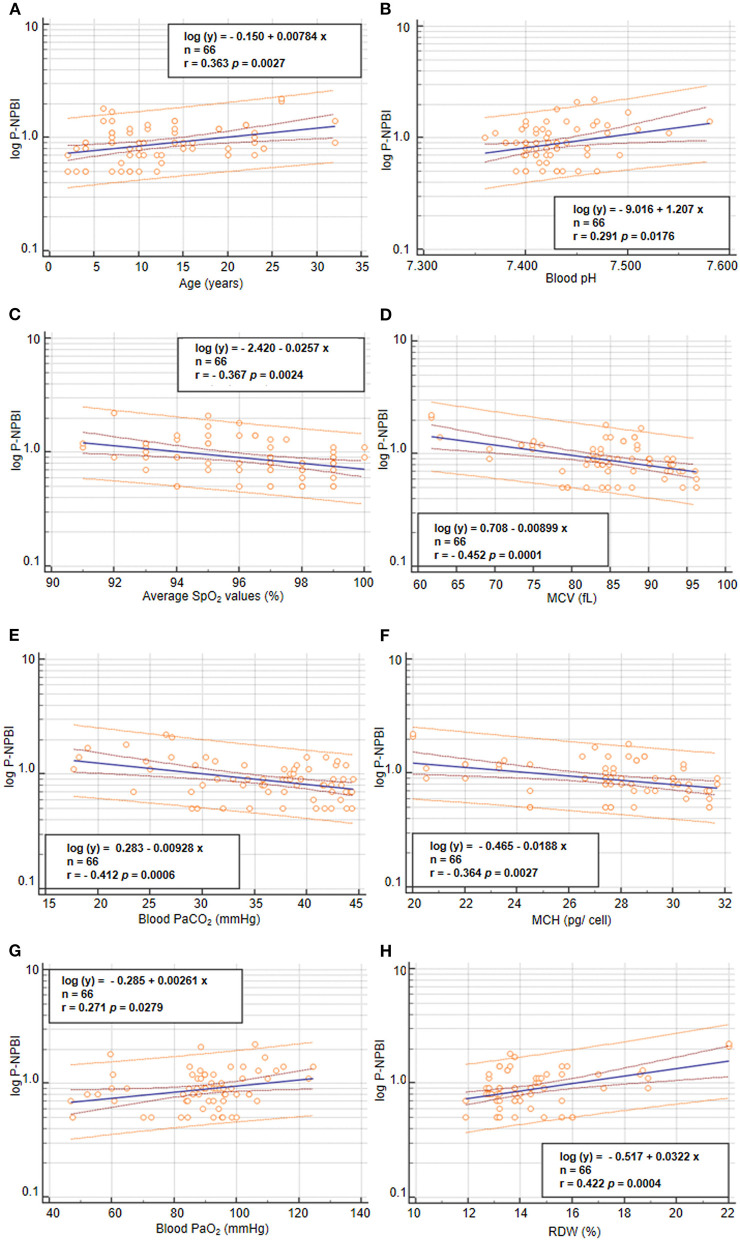
Correlations between P-NPBI and **(A)** age, **(B)** blood pH, **(C)** average SpO_2_, **(D)** MCV, **(E)** blood PaCO_2_, **(F)** MCH, **(G)** blood PaO_2_, and **(H)** RDW in the population with RTT (*n* = 66). P-NPBI concentrations are log-transformed to fit normal distribution. P-NPBI, plasma non-protein-bound-iron; SpO_2_, peripheral oxygen saturation; MCV, mean corpuscular volume; PaCO_2_, partial arterial pressure of carbon dioxide; MCH, mean cell hemoglobin content; PaO_2_, partial arterial pressure of oxygen; RDW, red cell distribution width. Inner dashed lines represent 95% confidence interval of regression. Outer lines represent 95% confidence interval of the predicted values.

Values of P-NPBI were positively correlated to age (*r* = 0.363, *p* = 0.0027), blood PaO_2_ values (*r* = 0.2707, *p* = 0.0279), and RDW (*r* = 0.422, *p* = 0.0004) (Figure 5). In contrast, P-NPBI values are inversely correlated to average SpO_2_ values (*r* = −0.367, *p* = 0.0024), blood pH (*r* = 0.291, *p* =0.018), blood PaCO_2_ values (*r* = −0.412, *p* = 0.0006), MCV (*r* = −0.454, *p* = 0.0001), and MCH (*r* = −0.364, *p* = 0.0027) (Figure 5).

Circulating levels of OS markers in the WA and SA subgroups are shown in Figure 4. In particular, P-NPBI levels were significantly elevated in the WA subgroup compared to the SA subgroup (*p* = 0.000054), whereas no significant differences were observed for IE-NPBI and plasma F_2_-IsoPs levels (*p* = 0.1813 and *p* = 0.9883, respectively).

### WA vs. SA as a Function of Epilepsy and AED Therapy

Based on presence of epilepsy and seizure frequency, no statistical differences were observed between the WA and SA subgroups (*p* = 0.2207 and *p* = 0.3462, respectively). In particular, 11/17 (64.7%) patients with RTT from the WA subgroup (WA^epi+^) vs. 39/49 (79.6%) patients with RTT from the SA subgroup (SA^epi+^) exhibited seizures. Among the WA^epi+^group, 2/11 (18.2%) had seizure frequency < monthly, 2/11 (18.2%) weekly to monthly, 5/11 (45.5%) weekly, and 2/11 (18.2%) daily to weekly.

Seizure frequency in the SA^epi+^ subgroup was as follows: 12/39 (30.8%) had seizure frequency of less than monthly, 4/39 (10.3%) weekly to monthly, 9/39 (23.1%) weekly, and 14/39 (35.9%) daily to weekly.

No statistical difference was observed for AED treatment (i.e., mono vs. multitherapy) between the WA and SA groups (RTT patients with WA: *n* = 5 AED mono- and *n* = 6 AED multi-therapy vs. RTT patients with SA: *n* = 19 AED mono- and *n* = 18 AED multi-therapy, *p* = 0.734).

In the WA^epi+^ group, the AED-treated patients were 10/11 (90.9%) on CBZ, 1/11 (9.1%) VPA, 4/11 (36.4%) TPM, and 2/11 (18.2%) PB. None in the WA^epi+^ group was on LEVET or CLB therapy. On the other hand, for the SA^epi+^group, 37/39 (94.9%) patients with RTT were on AED therapy, whereas 2/39 (5.1%) had no AED specific treatment. In particular, 32/37 (94.9%) were on CBZ, 7/37 (18.9%) VPA, 6/37 (16.2%) TPM, 4/37 (10.8%) LEVET, 2/37 (5.4%) CLB, and 6/37 (16.2%) PB. No significant differences were observed regarding AED therapy between the WA^epi+^and SA^epi+^ groups (*p* ≥ 0.1518).

Significant differences were observed for cardiorespiratory monitoring variables in patients with WA- and SA-RTT as a function of seizures, but with the single exception of total desaturation episodes (Kruskal -Wallis test, *p* = 0.1962). AHI >15 (chi-square test, *p* = 0.0099), total breath holding episodes (Mann-Whitney U-test, *p* = 0.0066), average SpO_2_ values (Mann-Whitney U-test, *p* = 0.0009), and nadir SpO_2_ values (Mann-Whitney U-test, *p* = 0.0028) being statistically different. Indeed, statistical differences for AHI > 15 were observed in the WA^epi−^vs. SA^epi+^groups (*p* = 0.0164) and in the WA^epi+^vs. SA^epi+^groups (*p* = 0.014). Statistical differences were observed for total breath holding episodes in non-epileptic patients with RTT (median 18.5 episodes in WA^epi−^vs. 59 in the SA^epi−^group) and epileptic patients with RTT (median 34 in WA^epi+^vs. 86 in SA^epi+^group) as well as WA^epi−^ vs. SA^epi+^groups (Conover test, *p* < 0.05). Similar results were also observed for average SpO_2_ values in epileptic patients with RTT (median 94% in WA^epi+^vs. 97% in the SA^epi+^group). Statistical differences were also observed between WA^epi−^ vs. SA^epi+^ (median 95% vs. 97%), and WA^epi+^ vs. SA^epi−^ (median 94 vs. 96.5%) (Conover test, *p* < 0.05). For nadir SpO_2_, values close to physiological range were recorded in both the WA^epi−^and WA^epi+^ subgroups (median 85.5 and 89%, respectively), which were statistically different from that observed in the SA^epi+^ group (72%, Conover test *p* < 0.05).

No statistical differences were observed in the populations with WA and SA for normalized respiratory rate (Mann-Whitney U-test, *p* = 0.837), blood pH (Mann-Whitney U-test, *p* = 0.4860), PaCO_2_ (Mann-Whitney U-test, *p* = 0.3828), PaO_2_ (Mann-Whitney U-test, *p* = 0.8198), standard PaO_2_ (Mann-Whitney U-test, *p* = 0.7327), ${\text{HCO}}_{3}^{-}$ (Mann-Whitney U-test, *p* = 0.5253), and BGA pattern interpretation (chi-squared test, *p* = 0.5746) as a function of seizures.

As it concerns red blood cell parameters, no statistical differences were observed for all the examined variables (i.e., Hb *p* = 0.461, MCV *p* = 0.122, MCH *p* = 0.069, MCHC *p* = 0.605, and RDW *p* = 0.399, Mann-Whitney U-test) in the populations with WA and SA as a function of seizures.

Significant differences were observed in the WA and SA groups for two circulating OS markers, P-NPBI (Mann-Whitney U-test, *p* = 0.00036) and plasma F_2_-IsoP levels (Mann-Whitney U-test, *p* = 0.0056) but not for IE-NPBI (Mann-Whitney U-test, *p* = 0.5321) (Supplementary Figures 8–10). In particular, P-NPBI levels were significantly higher in the WA^epi−^ and WA^epi+^ groups than in the SA^epi−^ and SA^epi+^groups (Conover test *p* < 0.05). Statistical differences were observed in patients with non-epileptic RTT (median 1.4 nmol/ml in WA^epi−^ vs. 0.8 in the SA^epi−^ subgroup) and in epileptic patients (median 1.2 nmol/ml in WA^epi+^ vs. 0.8 in the SA^epi+^ group). A statistical difference was observed for plasma F_2_-IsoP levels in the SA group (median 69.65 pg/ml in SA^epi−^ vs. 56.7 pg/ml in the SA^epi+^ group) as well as in the WA^epi+^ group vs. the SA^epi−^ group (Conover test, *p* < 0.05).

### ROC Curve and Stepwise Multiple Regression Models

**Figure 6:**
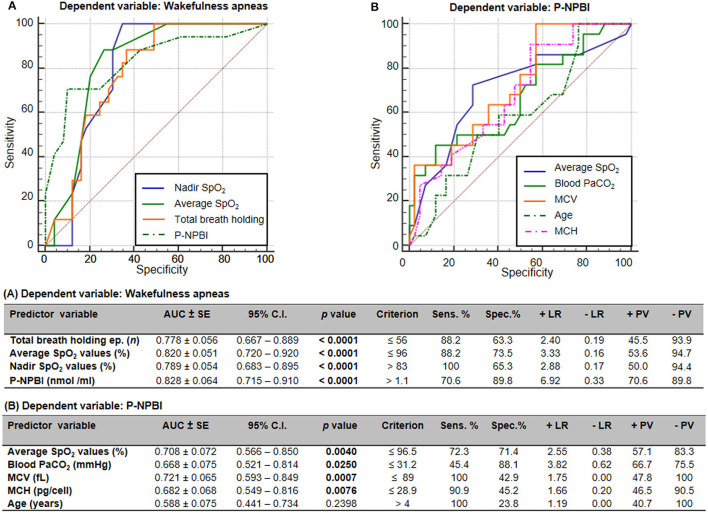
Receiver operating characteristic (ROC) curves analysis for **(A)** wakefulness apneas and **(B)** P-NPBI as classifying variables. AUC, area under the curve; SE, standard error; Sens, sensitivity; Spec, specificity; +LR, positive likelihood ratio; –LR, negative likelihood ratio; +PV, positive predictive value; –PV, negative predictive value; SpO_2_, peripheral oxygen saturation; P-NPBI, plasma non-protein-bound iron; PaCO_2_, partial arterial pressure of carbon dioxide; MCV, mean corpuscular volume; MCH, mean cell hemoglobin. Bold characters indicate statistically significant differences.

Receiver operating characteristic (ROC) curves indicated that total breath holding episodes, average SpO_2_ values, nadir SpO_2_ values, and P-NPBI values significantly discriminate WA from SA in RTT (Figure 6A). Furthermore, average SpO_2_ values, PaCO_2_ values, MCV values, MCH values, and age were able to discriminate higher circulating P-NPBI (Figure 6B).

**Figure 7:**
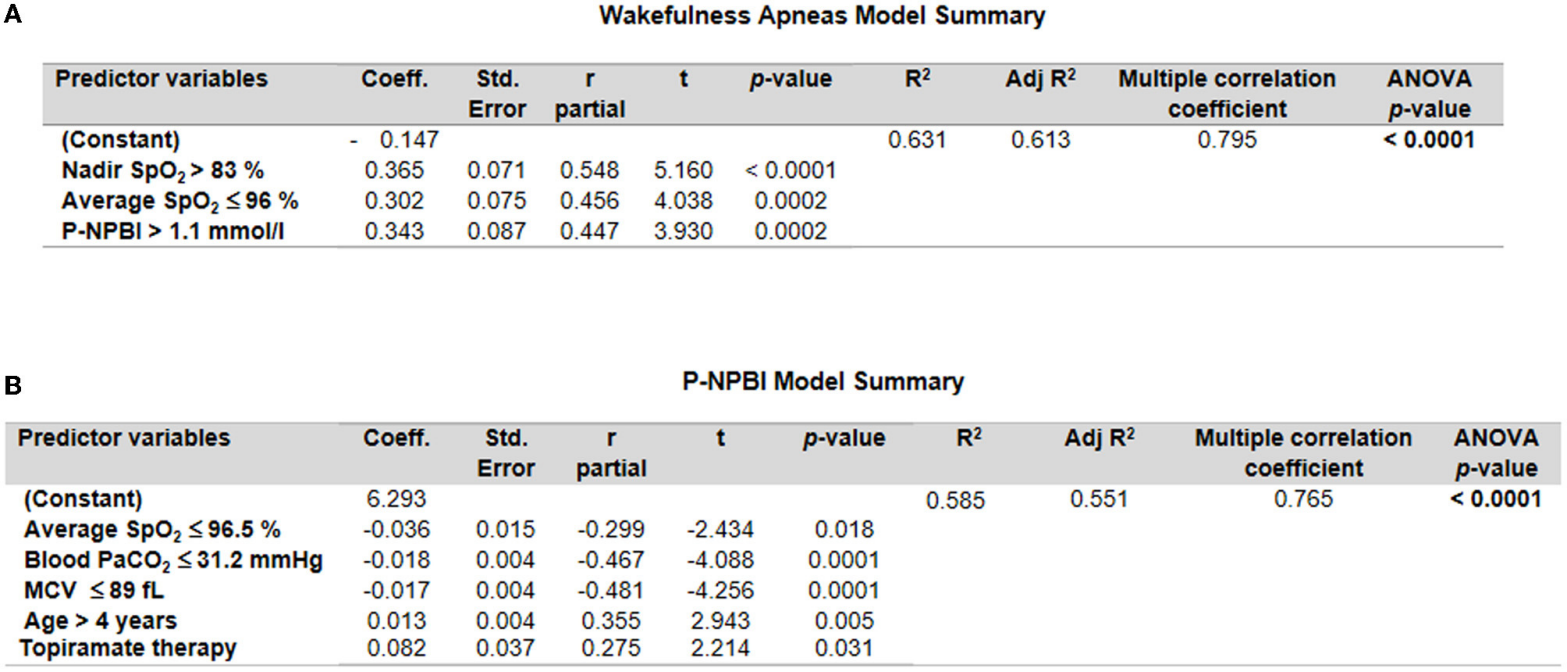
Multiple stepwise regression analysis models for **(A)** wakefulness apneas and **(B)** P-NPBI as dependent variables in the examined patients with RTT. Cut-off values were calculated by ROC curve analysis.

A multiple stepwise linear regression model (WA as the dependent variable) showed as predictor variables nadir SpO_2_, average SpO_2_, and P-NPBI (adjusted *R*^2^ = 0.613 multiple correlation coefficient = 0.795, *p* < 0.0001; variable not included in the model: total breath holding episodes) (Figure 7A).

An additional multiple stepwise linear regression model was investigated by selecting P-NPBI as the dependent variable. Although the red blood cell variable MCV was not statistically different between the WA and SA subgroups, this variable was selected among the possible predictor variables given the statistical significance of the ROC curve analysis (*p* = 0.0007). The model identified average SpO_2_, PaCO_2_, MCV, age, and topiramate therapy as significant predictors (adjusted *R*^2^ = 0.551, multiple correlation coefficient = 0.765, *p* < 0.0001) (Figure 7B).

## Discussion

Sleep disorders (1–3), breathing abnormalities (14–20), and redox imbalance (29–32) are widely recognized features of RTT. Awake breathing dysfunction is recognized as one of the most prevalent comorbidities of RTT, even more frequently than epilepsy (28). Sleep research with several RTT animal models (i.e., mice, cynomolgus monkeys, and *Drosophila*) indicates compromised sleep quality with highly fragmented sleep and distinct differences in daily sleep/wake cycle and circadian rhythm, thus mimicking the sleep abnormalities observed in patients with RTT (46). Breathing abnormalities in RTT models have been linked to non-rapid eye movement sleep (46). Previous clinical studies with patients with RTT indicate that sleep problems are reported in 80% cases, consisting of irregular sleep/wake patterns with increased sleep latencies or delayed/advanced sleep onset, excessive daytime sleep, and frequent nocturnal awakening (11, 47, 48). In addition, case studies showed that frequent arousals and low sleep efficiencies are characteristics of RTT (16). Many of these sleep problems in RTT appear to be consistent with an underlying problem in the circadian regulation of sleep.

In this study, where RTT patients with symptomatic respiratory dysfunction were selected exclusively, we investigated the relationships of breath holding episodes recorded during the sleep-wakefulness cycle with clinical variables, *MECP2* mutation type, cardiorespiratory monitoring, blood gas balance, red blood cell parameters, and circulating oxidative stress markers.

In our RTT cohort, an excess of patients of pediatric age, disease stage III, and early truncating mutations were related to clinically evident breathing abnormalities either during sleep or wakefulness.

Although about half of our patients were in stage III of the disease, our observations confirm that breathing abnormalities are already present during the pediatric age. As previously reported, awake breathing abnormalities have been reported to follow an age-dependent pattern, with hyperventilation and breath holding starting by age 5 in two-thirds of the RTT patients, often remitting over the lifespan (28). The RTT-specific BMI *z*-scores of our population with documented breath holding episodes showed a normal distribution, thus supporting prior observations (28). It has been suggested that individuals with a normal weight for RTT are more likely to experience breath holding episodes. Nevertheless, subjects with both normal and high body mass index for RTT appear to be equally likely prone to hyperventilation (28).

To date, a large number of clinical and experimental studies have been focusing on breathing disorders in RTT (49) variably featuring breath holding episodes, apneas, apneusis, hyperventilation, rapid shallow breathing, and spontaneous Valsalva maneuvers (50). The wide spectrum of respiratory disorders detectable in patients with RTT has been historically credited to brainstem immaturity and/or cardiorespiratory autonomic dysautonomia (50, 51). However, as the pathogenesis of the respiratory dysfunction in RTT appears far from being completely understood, alternative or complementary hypotheses can be formulated (30).

Highly irregular respiratory rhythm, particularly during daytime, is considered a key symptom of RTT (22, 23, 50). Cumulating evidence indicates a predominantly hyperventilatory pattern with breath holding/obstructive apneas or Valsalva breathing against closed airways during wakefulness (23, 52, 53). Breath holding/obstructive apneas in RTT patients are often confused with central apneas, where distinct neurological mechanisms exist (14–16, 24, 26, 50, 54–59). Furthermore, a high incidence of Obstructive Sleep Apnea Syndrome (OSAS) with large variability in severity has been reported in patients with RTT and respiratory disturbances (15, 16, 19).

In this study, the whole RTT population examined showed high prevalence of obstructive apneas compared to the central and mixed ones, thus confirming our prior observations (32). OSAS are relatively common in the general pediatric population, with estimated prevalence between 1 and 4% (60, 61). Adenotonsillar hypertrophy is a widely recognized as a major significant contributor to OSAS for otherwise healthy children (62), with the American Academy of Pediatrics indicating adenotonsillectomy as a first-line treatment for pediatric OSAS (63). According to latest guidelines, the general incidence of OSAS in the pediatric population is about 2% (63). OSAS are more common in males than in females (60), and in most children around 2–8 years of age because of the relative size of the lymphatic tissue of the upper airways (64). However, OSAS related to adenotonsillar hypertrophy do not seem to be a specific feature of RTT (18).

Although drug-induced sleep endoscopy (DISE) is certainly a quite promising diagnostic procedure for investigating upper airway mechanics during sleep in pediatric and adult populations showing obstructive sleep apneas (65, 66), this technique was, unfortunately, not performed on our RTT population because of lack of local ethical approval. This point may be a possible study limitation, although we should stress that causes of apneas and upper airway mechanics during sleep were not a primary focus of this study.

While some authors report sleep-related breathing disorders as non-clinically relevant (14), impaired sleep structure and breathing patterns have been characterized in RTT patients by polysomnography and EEG studies (16, 18–20, 58, 67). On the other hand, disorders of respiratory control during wakefulness are well-recognized (14, 23, 28). The results of this study confirm the occurrence of breath holding episodes in the patients with RTT at different ages and disease stages either during the awake or the sleeping state. However, the lack of simultaneous EEG recording during the cardiorespiratory monitoring could represent a methodological limitation to the present study. The link between respiratory dysfunction and disease severity indicates its clinical relevance.

Dysregulation of autonomic circuits is a key feature of RTT (68–70), with diurnal variation in the autonomic regulation and shift toward sympathetic activation and/or parasympathetic inactivation (71).

Although the small sample size prevents general conclusions, our findings suggest a link between specific types of *MECP2* mutations (i.e., R255X, large deletions, and R106W) and cardiorespiratory monitoring findings. In particular, our observation of a positive link between recorded total breath holding episodes and R270X *MECP2* mutations appears in line with prior observations by Carroll et al. (71) of major alterations in amplitude and phase of autonomic balance diurnal patterns observed in patients with RTT with early truncating *MECP2* mutations. In contrast, a prior natural history study on a large RTT cohort has evidenced breathing dysfunction not being related to specific mutations with the presence of *MECP2* mutation as the only genetic factor being associated with breathing dysfunction (28). A possible explanation of the discordance with our findings could be related to either our different selection criteria (i.e., inclusion of patients with clinical evidence of breathing disturbances) or sample size effects. However, rather than examining the natural course of respiratory abnormalities in the disease, our primary aim was to assess the clinical relevance of apneas during the sleep-wakefulness cycle in the population with RTT and their possible clinical impact.

Our observation of an inverse relationship between average SpO_2_ and age confirms the existence of an age-dependent pattern (28), even if in our selected cohort breathing dysfunction does not fade with advancing age and/or disease stage.

The majority of RTT patients suffer from severe cardiorespiratory symptoms (57, 72–74), possibly accounting for a fraction with a 300-fold increased risk of sudden death (68, 75) and a likelihood of survival of 70% at age 45 years (76). Respiratory-related reflexes appear to be exaggerated (77, 78), lacking distinct forms of reflex plasticity (79). There is growing evidence that a progressively developing neurochemical dysfunction and balance of neuronal excitation and inhibition skewed toward hyperexcitability in cardiorespiratory pontomedullary brain stem areas (79–83) could cause the reported deficits (84).

Accordingly, several studies with *Mecp2*-mutant rodent models report increased excitability of neurons from cranial nerve V (85, 86). More recently, a study on the hypoglossal nucleus (XII) and dorsal motor nucleus of vagus (DMNV, X) cranial nerves demonstrates dual synaptic GABAA-ergic and glycinergic synaptic inhibitions, thus suggesting that these neurons rely more on glycinergic synaptic inhibition and possibly contribute to the breathing abnormalities observed in mouse models of RTT (87). Likewise, similar results are found by Yoshikawa (88) and Yamanouchi (89), reporting cortical hyperexcitability in patients with RTT. Furthermore, a neuropathological study (90) on a small population of patients with RTT with mean age of 10.8 ± 6.3 years suggests serotonin transporter abnormality in the DMNV, possibly underlying the autonomic dysfunction observed in RTT.

Interestingly, in the examined population with RTT, no clinical signs related to dysfunction(s) of cranial nerves, in particular V, VII, and XII, were detectable on a careful independent neurological examination by three expert MDs (JH, RC, and CDF), which is in line with prior observations by Armstrong (91).

Respiratory disturbances in RTT could also lead to potentially life-threatening systemic hypoxia. Our prior observations indicate that pulmonary gas exchange abnormalities can play a role in determination of systemic hypoxia in girls with RTT (30, 32), with terminal bronchioles as a likely major target in the disease (34).

Although of key importance in order to objectify the respiratory dysfunction linked to the disease, arterial blood gas measurements are unusually reported in patients with RTT (30, 32, 55, 92).

The relationship between hypoxia and *MECP2* loss of function is certainly complex. Experimental studies on mouse models of the disease indicate that Mecp2 is necessary for survival (93) and a correct hypoxia response (94). Arterial blood gas analysis data on our RTT population indicate the co-existence of a chronic hyperventilation condition with subsequent hypocapnia and respiratory alkalosis.

In particular, a baseline hypoxia in about one quarter of RTT patients exhibiting clinical evidence of apneas/breath holdings was observed. These findings are in line with our prior data indicating a state of subclinical hypoxia in the majority of patients with RTT (30, 32).

Several studies have attempted to evaluate the role of hypoxia using RTT experimental models (31, 78, 93–103). Exposure to hypoxic conditions has been induced in experimental models (i.e., mice, rats, cell cultures, and/or histologic brainstem/hippocampal sections). However, the degree, nature of hypoxic *noxae* (i.e., reduced inspired/environmental fraction of oxygen, CO_2_- or chemically-induced hypoxia), and duration of hypoxic challenge are extremely variable (94–97, 99, 100). Therefore, comparing the findings appears to be quite difficult. Nevertheless, exaggerated response/susceptibility to hypoxia seems a consistently shared feature (78, 84, 98, 99, 101, 103, 104). In addition, more than one-third of our patients with RTT showed hypocapnia. Therefore, chronic hypoxia with hypocapnia seems to be a common shared feature among patients with clinically evident breathing disturbances. These findings indicate a role for compensatory hyperventilation following a chronic hypoxia condition.

It is well-known that to maintain O_2_ and CO_2_ homeostasis in the blood and tissues, the respiratory central pattern generator must respond to chemosensory cues. Under conditions of chronic intermittent hypoxia, repeated peripheral chemoreceptor input, as mediated by the nucleus of the solitary tract, induces plastic changes in respiratory circuits altering baseline respiratory and sympathetic motor outputs, and resulting in chemoreflex sensitization, active expiration, and arterial hypertension (104). Interestingly, respiratory depression follows hypocapnia in RTT mouse models (95). This phenomenon would likely account for our findings on individuals with RTT of depressed respiratory rate *z*-scores despite the presence of marked hypoxia.

Respiratory alkalosis is likely to be compensated by renal excretion of ${\text{HCO}}_{3}^{-}$, i.e., *via* compensatory metabolic acidosis according to the Henderson and Hasselbalch Equation modifying hemoglobin affinity for O_2_ because of Bohr and Haldane effects (105, 106). This compensatory mechanism is likely to account for the observed decrease in ${\text{HCO}}_{3}^{-}$ detectable in the overwhelming majority of the examined patients with RTT.

In contrast, arterial hypertension is not usually observed in patients with RTT. Indeed, in our personal extended database, mean systolic blood pressure and diastolic blood pressure values correspond to 0.95 ± 0.1 (value range 0.81–1.24) and 0.92 ± 0.12 (0.73–1.18) multiple of medians compared to age- and gender-matched healthy control subjects (individual data not shown). In line with the well-known cardiac autonomic dysfunction (68, 70, 71), resting awake heart rate values were found to be increased, corresponding to 1.1 [0.98–1.30] (0.90–1.74) multiple of medians for age- and gender-matched healthy population (individual data not shown; JH, CDF, VS, RC, personal observation).

Erythrocytes appear to be key cells in RTT (33, 107, 108). We have previously reported the high prevalence of atypical shape for circulating red blood cells (i.e., leptocytosis) (33) with increased 4-hydroxynonenal protein adducts in erythrocyte cytoskeleton plasma membrane proteins (108). Despite significant alterations in antioxidant defense capability, erythrocytes from patients with RTT do not show intrinsic differences in terms of neither Hb-binding affinity nor in O_2_ exchange processes compared to control erythrocytes (109).

In this cohort of patients with RTT, asymptomatic anemia was detected in about one-third of the whole population. Among the selected RBC variables, Hb and MCHC appear to be significantly decreased compared to a general population with RTT (Hb 13.2 ± 1 g/d, *p* =0.036; MCHC 33.8 ± 1.09 g/dl, *p* = 0.0003; individual data not shown; JH, CDF, VS, RC, personal unpublished observations).

These data are in apparent contrast with prior research on an RTT mouse model (*Mecp*2/y mice) where increased hematocrit and elevated hypoxia-inducible factor (HIF)-1 expression levels throughout the brain have been reported as signs of systemic adaptation to intermittent hypoxic episodes (98). Nevertheless, iron-deficiency anemia has been reported in about one-eighth of the general population with RTT (110), a proportion apparently not different from ours (Fisher's test, *p* = 0.109).

The origin of anemia in RTT could be related to reduced iron intake (111, 112) with consequently reduced iron stores (113).

Accumulating evidence reveals that the lungs are a key organ in iron homeostasis. Regulation of iron homeostasis in mammalian lung is tightly controlled (iron content range: 0.4–0.9 mg/g lung tissue) (114) in order to maintain proper lung function and to adapt to changes in iron needs (115).

Iron's critical role is explained by its potential to fluctuate between oxidation states, mainly between divalent ferrous (Fe^2+^) and trivalent ferric (Fe^3+^) iron (116). However, this chemical property as a transition metal makes free iron very reactive and potentially toxic. Iron catalyzes the production of reactive oxygen species (ROS) *via* the Fenton and Haber-Weiss reactions (116). Exposure to these highly reactive radicals damages lipids, nucleic acids, and proteins, causing cell and, thus, tissue damage.

Our data confirm increased circulating OS marker levels, and are in line with prior studies (30, 32, 109). OS markers comprised two forms of redox active-iron, i.e., P-NPBI and IE-NPBI, thus confirming prior findings (30, 32). Although the possible role of epileptic activity in OS status (117–119) remains a fascinating subject of investigation, some studies report controversial findings. In our study, a relationship between increased P-NPBI levels and TPM, a widely used AED with multiple mechanisms of action (120), was observed. Some prior studies report a protective effect of TPM on oxidative injury (121–123), while other reports suggest no effects on OS status (124, 125). To date, no information on the effects of TPM on circulating redox active iron is available. Its pharmacological properties include modulatory effects on Na^+^ channels, GABA-A receptors, and glutamate receptors of the α-amino-3-hydroxy-5-methyl-4-isoxazolepropionic acid (AMPA)/kainate type (126). This favorable combination of mechanisms of action makes TPM an ideal candidate for antiepileptogenesis in the clinical field. Intriguingly, TPM is also used in prophylaxis of chronic migraine (127), a condition in which iron overload in the periaqueductal gray matter (128), red nucleus, and basal ganglia structures has been reported (127, 129). Overall, our findings, combined with the findings of published literature, suggest a hitherto unrecognized link between TPM and free redox active iron. This point is in need of further exploration.

Although autonomic dysregulation in RTT has been the focus of extensive investigation during the sleep-awake cycle (27, 71), little is known on possible differential effects of RTT-related breathing abnormalities during the sleep-wakefulness cycle on acid-base balance, erythrocyte variables, and pro-oxidant status.

Our data indicate that breathing abnormalities are prevalent both in the sleep and wakefulness states in a population with RTT with clinically evident apneas. Overall AHI > 15, higher total breath holding episodes, and lower nadir SpO_2_ were more likely in the subpopulation with predominant SA, whereas lower average SpO_2_ was related to WA. These findings indicate that the autonomic dysregulation in RTT is closely linked to the sleep-wakefulness cycle, which is in line with recent observations by Ramirez et al. (27). While no significant relationships were observed with acid-base, and baseline respiratory rate (*z*-scores), slightly lower MCH content was correlated to patients with respiratory abnormalities mainly linked to the awake state. Although no plausible explanation is available to date, these findings stimulate a more in-depth investigation on the relationship between iron status and breathing disturbances in patients with RTT.

In addition, for the first time, our study indicates a different impact of wakefulness apnea episodes on plasma free redox-active iron compared to sleep apneas in patients with RTT. In this study, a stepwise multiple linear regression model indicated a significant relationship between P-NPBI and average SpO_2_, blood PaCO_2_, red blood cell MCV, age, topiramate therapy. In addition, WA was also related to circulating P-NPBI.

While iron is essential for aerobic life, poor or altered iron handling leads to adverse effects related to oxidant production, altered redox signaling, and altered cellular fate, including remodeling. Unfortunately, lack of cooperation in RTT hampers more in-depth studies on the redox active pulmonary pool (i.e., bronchoalveolar lavage fluid). However, a specific pro-oxidant pool of iron (i.e., free or loosely bound ions, which are redox-active/catalytic for damaging oxidant production) is also measurable in exhaled breath condensate (130). It has been previously demonstrated that retention of iron in the airways and lungs may contribute to manifestations of OS and damage in chronic obstructive pulmonary disease (131), acute respiratory distress syndrome (132), and coronavirus disease-2019 (COVID-19) (132).

Overall, our data indicate that this redox-active iron form is a relevant molecular factor in the pathophysiology of respiratory abnormalities in RTT, and suggest that plasma redox-active iron could represent a potential novel therapeutic target by deepening our current understanding of the apparently unique respiratory pathophysiology of patients affected by this rare neurodevelopmental disorder.

## Data Availability Statement

The raw data supporting the conclusions of this article will be made available by the authors, without undue reservation.

## Ethics Statement

The studies involving human participants were reviewed and approved by Ethical Committee of Siena University Hospital (Azienda Ospedaliera Universitaria Senese, Siena, Italy). Written informed consent to participate in this study was provided by the participants' legal guardian/next of kin.

## Author Contributions

CDF and SL: conceptualization and writing of the original draft. JH and LC: funding acquisition. JH: subject enrollment. JH, RC, and CDF: collection of clinical information. JH and CDF: RCSS severity scoring assessment. MR: evaluation and interpretation of the cardiorespiratory/polygraphy data. SL: P-NPBI and IE-NPBI assays. CS: plasma F_2_-IsoP assays. LC: supervision of the oxidative stress laboratory procedures. LB and VS: clinical database. CDF: data analysis. LC, CS, and MR: draft of sections of the manuscript. All the authors contributed to manuscript revision, read, and approved the submitted version.

## Funding

This study was partly funded by the Tuscany Region (Italy), Bando Salute 2009: Antioxidants—omega-3 polyunsaturated Fatty Acids, lipoic acid—supplementation in Rett syndrome: A novel approach to therapy, RT No. 142.

## Conflict of Interest

The authors declare that the research was conducted in the absence of any commercial or financial relationships that could be construed as a potential conflict of interest.

## Publisher's Note

All claims expressed in this article are solely those of the authors and do not necessarily represent those of their affiliated organizations, or those of the publisher, the editors and the reviewers. Any product that may be evaluated in this article, or claim that may be made by its manufacturer, is not guaranteed or endorsed by the publisher.
