# Supplementary material for: Breathing Abnormalities During Sleep and Wakefulness in Rett Syndrome: Clinical Relevance and Paradoxical Relationship With Circulating Pro-oxidant Markers
Source: Front Neurol. 2022 Mar 29;13:833239. doi: 10.3389/fneur.2022.833239 (PMC9001904; doi:10.3389/fneur.2022.833239)
Supplement: Supplementary file 1 [file Image_1.pdf]

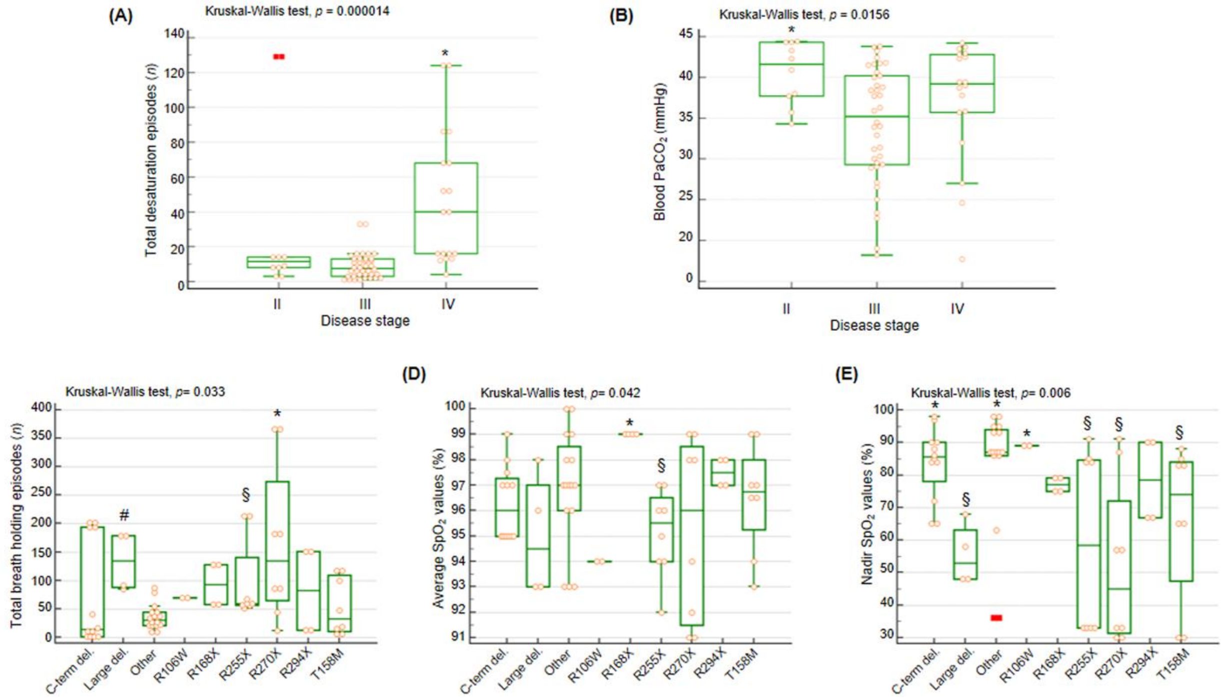

Supplementary Figure S1. (A) Total desaturation episodes as a function of disease stage. *Post-hoc* analysis (Conover test): * $p < 0.05$ *vs*. stage II and stage III. **(B)** Blood $PaCO_2$ as a function of disease stage. *Post-hoc* analysis (Conover test): * $p < 0.05$ *vs*. stage III and stage III. Total breath holding episodes **(C)**, average SpO_2 values **(D)**, and nadir SpO_2 values **(E)** categorized by *MECP2* mutation type. **(C)** Total breath holding episodes. *Post-hoc* analysis (Conover test): * *vs*. T158M, Other, C-term del.; § *vs*. Other, C-term del.; # *vs*. C-term del., Other. **(D)** Average SpO_2. *Post-hoc* analysis (Conover test): * *vs*. C-term del., Late del., Other, R106W, R255X, R270X, T158M. § *vs*. Other. **(E)** Nadir SpO_2. *Post-hoc* analysis (Conover test): * *vs*. Late del., R255X, R270X, T158M. § *vs*. Other, R106W. $PaCO_2$: partial arterial pressure of carbon dioxide; SpO_2: peripheral oxygen saturation; C-term del.: C-terminal deletions; Large del.: large deletions; Other: non-hotspot pathogenic *MECP2* mutations. Data are shown as box- and whisker-plots. Red rectangles indicate outlier data points.
