# Supplementary material for: Breathing Abnormalities During Sleep and Wakefulness in Rett Syndrome: Clinical Relevance and Paradoxical Relationship With Circulating Pro-oxidant Markers
Source: Front Neurol. 2022 Mar 29;13:833239. doi: 10.3389/fneur.2022.833239 (PMC9001904; doi:10.3389/fneur.2022.833239)
Supplement: Supplementary file 2 [file Image_2.pdf]

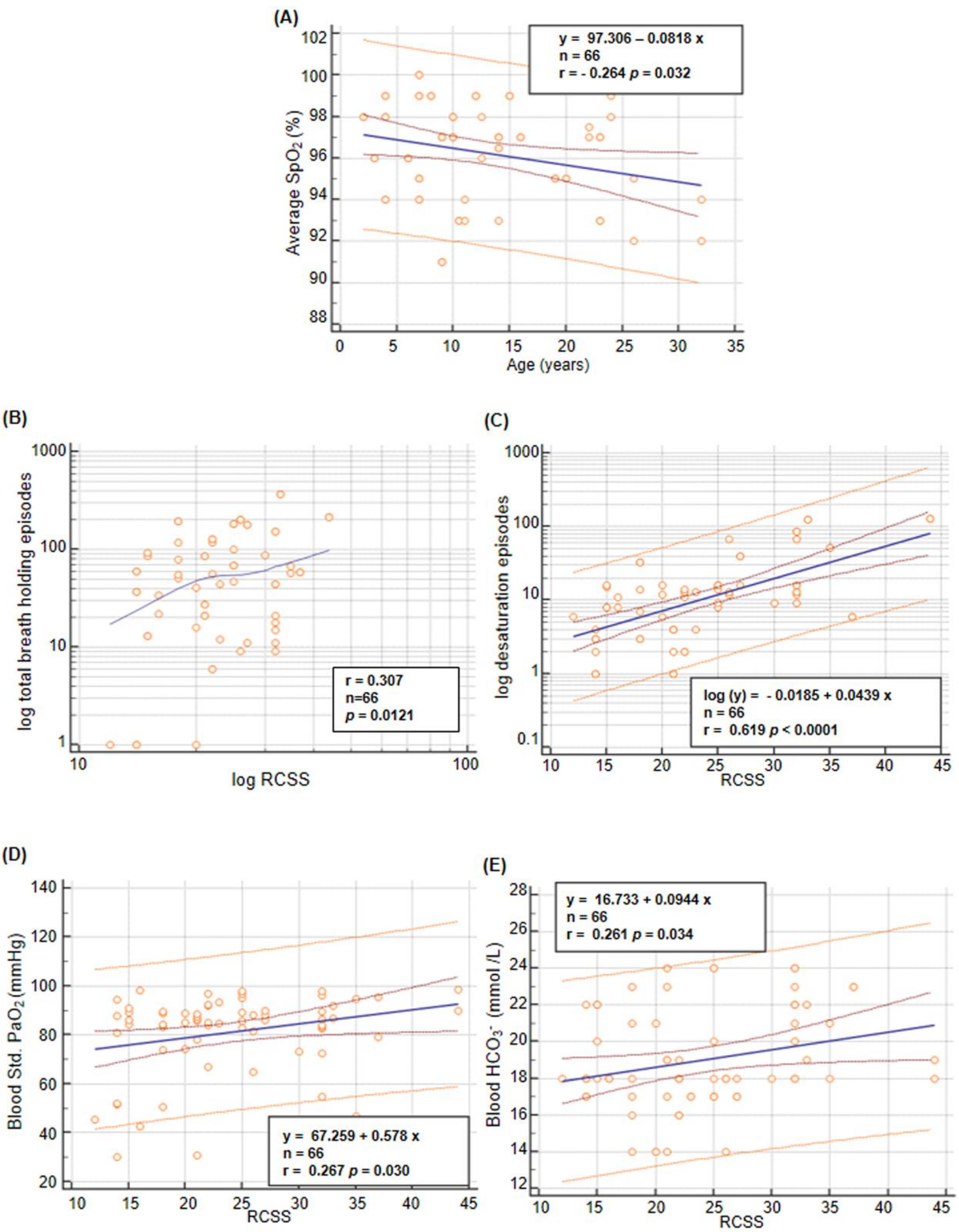

Supplementary Figure S2. Miscellaneous correlations between demographic, cardiorespiratory monitoring, blood gas analysis variables and disease severity. **(A)** Inverse linear correlation between average SpO_2 and age. **(B)** Scatter plot of total breathing holding episodes logarithmic-transformed variables. Trend line is shown by a LOESS (local polynomial regression) fitting curve. **(C)** Positive correlation between desaturation episodes and clinical severity (RCSS). Desaturation episodes were log-transformed to fit normal distribution. **(D, E)** Positive correlations between blood Std. PaO_2, blood HCO_3^- and clinical severity (RCSS). SpO_2: peripheral oxygen saturation; Std. PaO_2: standardized PaO_2 accounting for hypocapnia as calculated according to Sorbini et al. (40); RCSS: Rett Clinical Severity Score. Inner dashed lines represent 95% confidence intervals of regression. Outer lines represent 95% confidence intervals of the predicted values.
