# Supplementary material for: Breathing Abnormalities During Sleep and Wakefulness in Rett Syndrome: Clinical Relevance and Paradoxical Relationship With Circulating Pro-oxidant Markers
Source: Front Neurol. 2022 Mar 29;13:833239. doi: 10.3389/fneur.2022.833239 (PMC9001904; doi:10.3389/fneur.2022.833239)
Supplement: Supplementary file 3 [file Image_3.pdf]

(A) (B)

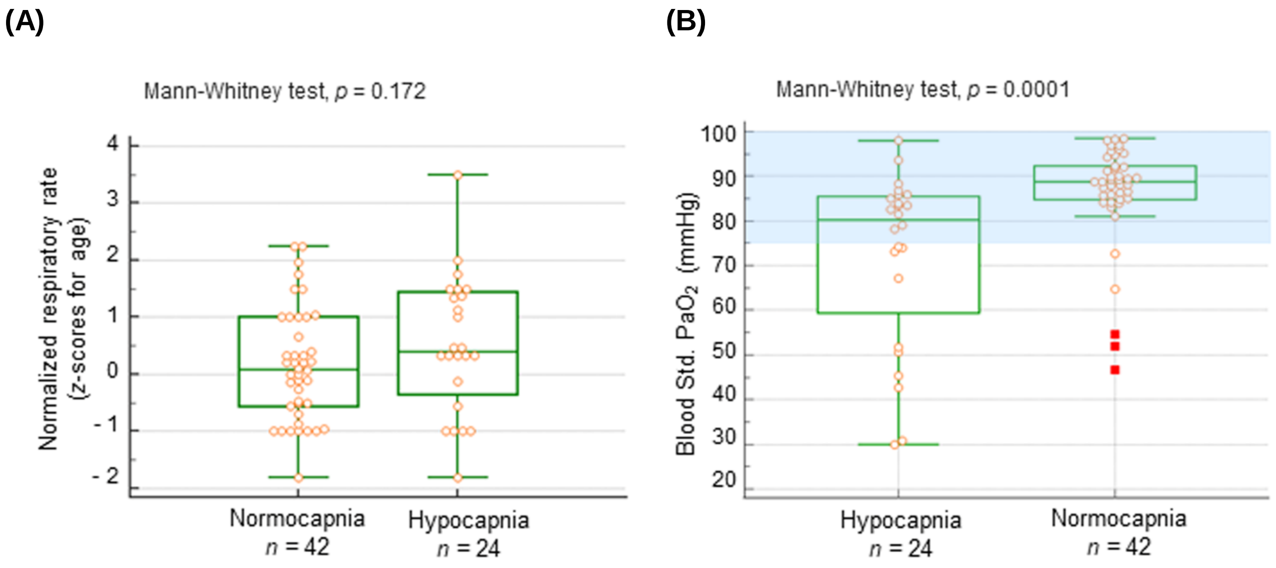

Supplementary Figure S3. (A) Normalized respiratory rate (z-scores for age) and **(B)** Blood Std. PaO_2 values as a function of normo- or hypocapnia (i.e., $PaCO_2 < 35$ mmHg) in the examined RTT population (n=66). Abbreviations: Std. PaO_2: standardized PaO_2 accounting for hypocapnia as calculated according to Sorbini et al. (41). Data are shown as box- and whisker-plots. Red rectangles indicate outliers.
