# Supplementary material for: Breathing Abnormalities During Sleep and Wakefulness in Rett Syndrome: Clinical Relevance and Paradoxical Relationship With Circulating Pro-oxidant Markers
Source: Front Neurol. 2022 Mar 29;13:833239. doi: 10.3389/fneur.2022.833239 (PMC9001904; doi:10.3389/fneur.2022.833239)
Supplement: Supplementary file 4 [file Image_4.pdf]

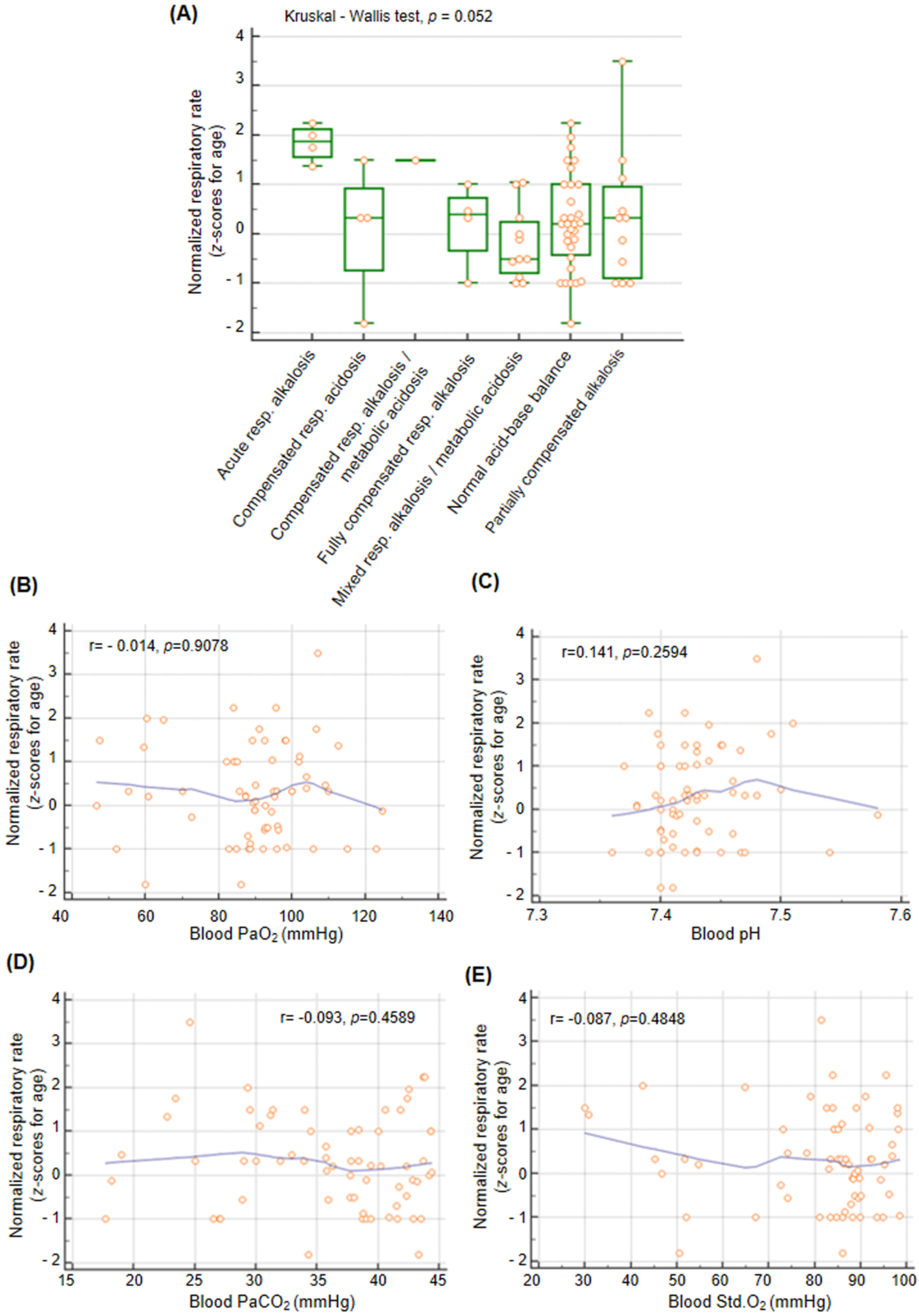

Supplementary Figure S4. (A) Distribution of normalized respiratory rate (z-scores for age) as a function of acid-base balance status. **(B-E)** Exploratory study on the relationships between normalized respiratory rate (z-scores for age), blood pH, and arterial blood gas parameters (PaO_2, Std. O_2, $PaCO_2$) in the whole RTT population (*n*=66). The trend line is shown by a LOESS (local polynomial regression) fitting curve. Abbreviations: Std. PaO_2: standardized PaO_2 accounting for hypocapnia as calculated according to Sorbini et al. (41); PaO_2: partial arterial pressure of oxygen; $PaCO_2$: partial arterial pressure of carbon dioxide
