# Supplementary material for: Breathing Abnormalities During Sleep and Wakefulness in Rett Syndrome: Clinical Relevance and Paradoxical Relationship With Circulating Pro-oxidant Markers
Source: Front Neurol. 2022 Mar 29;13:833239. doi: 10.3389/fneur.2022.833239 (PMC9001904; doi:10.3389/fneur.2022.833239)
Supplement: Supplementary file 5 [file Image_5.pdf]

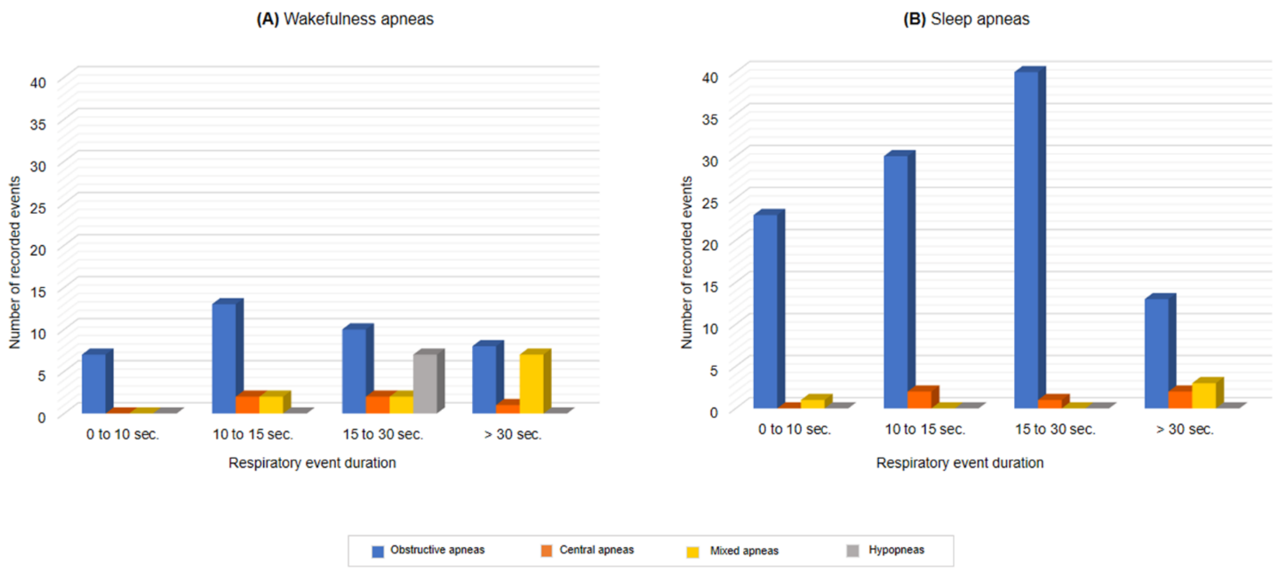

Supplementary Figure S5. Paradigmatic respiratory events distribution in RTT girls in the WA- and SA-subpopulation. **(A)** Respiratory events distribution in a WA-RTT patient (age 6 yr.s, disease stage III, *MECP2* genotype G252Xfs). **(B)** Respiratory events distribution in a SA-RTT patient (age 2 yr.s, disease stage II, *MECP2* genotype G269Afs, variant NM_004992.3). WA: wakefulness apneas. SA: sleep apneas
