# Supplementary material for: Breathing Abnormalities During Sleep and Wakefulness in Rett Syndrome: Clinical Relevance and Paradoxical Relationship With Circulating Pro-oxidant Markers
Source: Front Neurol. 2022 Mar 29;13:833239. doi: 10.3389/fneur.2022.833239 (PMC9001904; doi:10.3389/fneur.2022.833239)
Supplement: Supplementary file 6 [file Image_6.pdf]

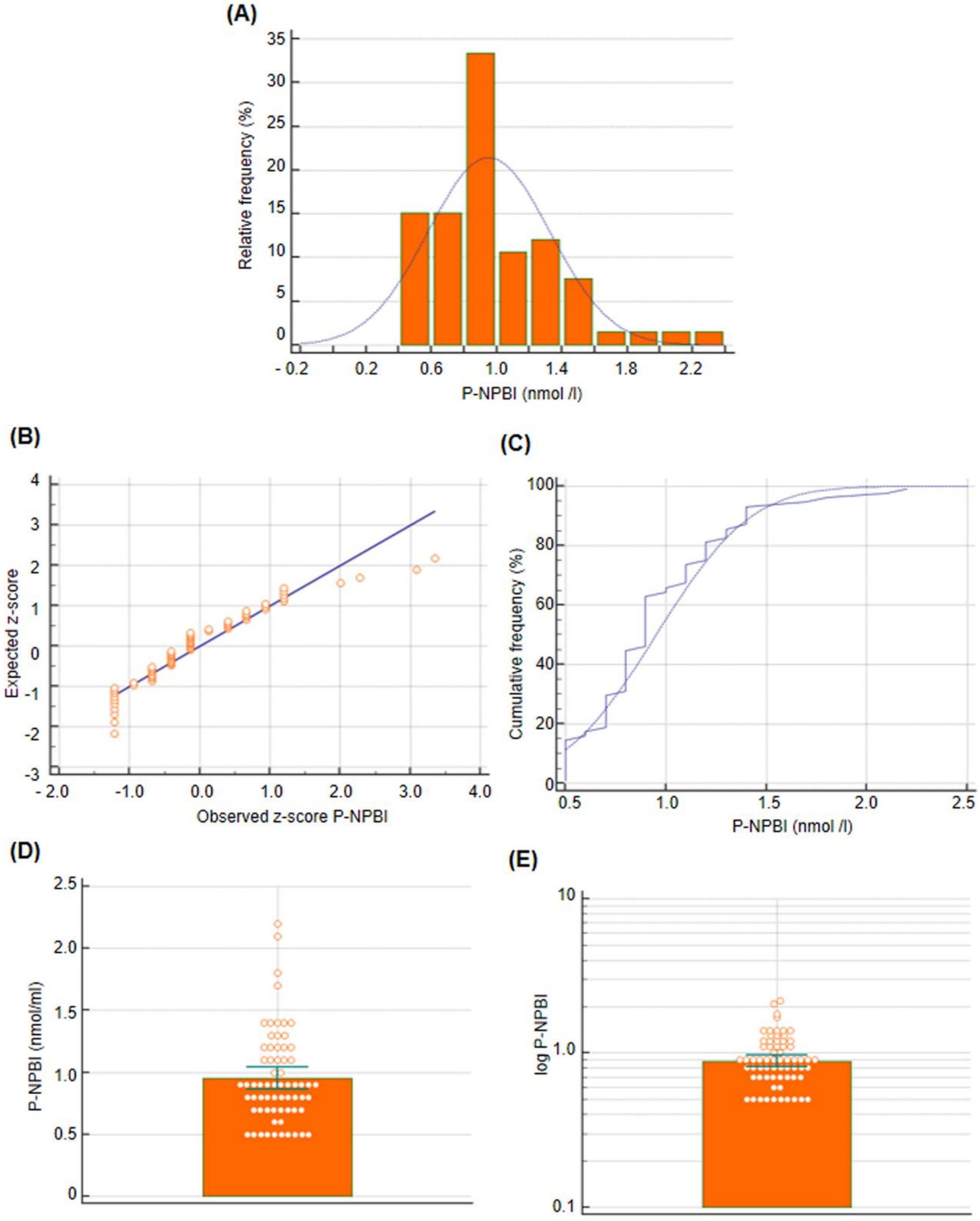

Supplementary Figure S6. (A) Redox active iron (P-NPBI) data distribution: histogram and relative frequency. Normal distribution is shown (dotted line). **(B)** P-NPBI normal plot: observed vs. expected z-scores. **(C)** P-NPBI cumulative frequency distribution (solid line). For reference, normal distribution is also shown (dotted line). P-NPBI dot plots: raw **(D)** vs. log transformed data **(E)**. P-NPBI, plasma non-protein-bound iron.
