# Supplementary material for: Breathing Abnormalities During Sleep and Wakefulness in Rett Syndrome: Clinical Relevance and Paradoxical Relationship With Circulating Pro-oxidant Markers
Source: Front Neurol. 2022 Mar 29;13:833239. doi: 10.3389/fneur.2022.833239 (PMC9001904; doi:10.3389/fneur.2022.833239)
Supplement: Supplementary file 7 [file Image_7.pdf]

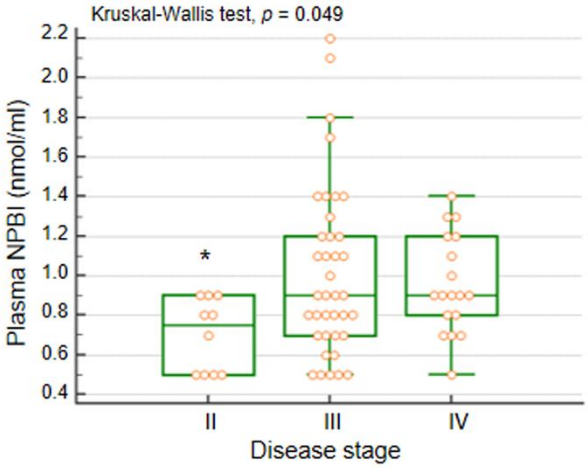

Supplementary Figure S7. P-NPBI values as a function of disease stage in the examined RTT population (n=66). P-NPBI: plasma non-protein-bound iron. Data are shown as box- and whisker-plots.
