# Supplementary material for: Breathing Abnormalities During Sleep and Wakefulness in Rett Syndrome: Clinical Relevance and Paradoxical Relationship With Circulating Pro-oxidant Markers
Source: Front Neurol. 2022 Mar 29;13:833239. doi: 10.3389/fneur.2022.833239 (PMC9001904; doi:10.3389/fneur.2022.833239)
Supplement: Supplementary file 8 [file Image_8.pdf]

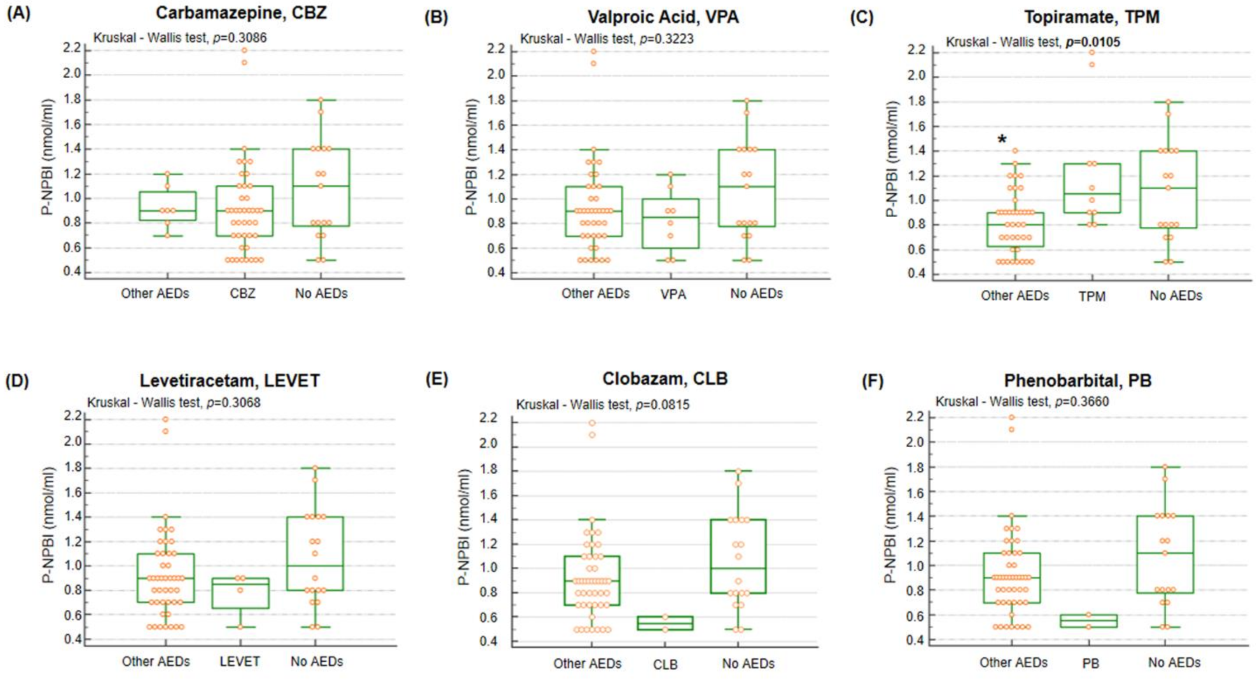

Supplementary Figure S8. P-NPBI levels as a function of antiepileptic drug therapy in the examined RTT population (n=66) **(A-F)**. No statistical differences were observed for CBZ **(A)**, VPA **(B)**, LEVET **(D)**, CLB **(E)**, and PB **(F)** treatment. **(C)** TPM treatment. *Post-hoc* analysis (Conover test): * $p < 0.05$ *vs*. TPM group and other AED group. P-NPBI: plasma non-protein-bound iron. CBZ: carbamazepine. VPA: valproic acid. TPM: topiramate. LEVET: levetiracetam. CLB: clobazam. PB: phenobarbital. Data are shown as box - and whisker-plots.
