# Supplementary material for: Breathing Abnormalities During Sleep and Wakefulness in Rett Syndrome: Clinical Relevance and Paradoxical Relationship With Circulating Pro-oxidant Markers
Source: Front Neurol. 2022 Mar 29;13:833239. doi: 10.3389/fneur.2022.833239 (PMC9001904; doi:10.3389/fneur.2022.833239)
Supplement: Supplementary file 10 [file Image_10.pdf]

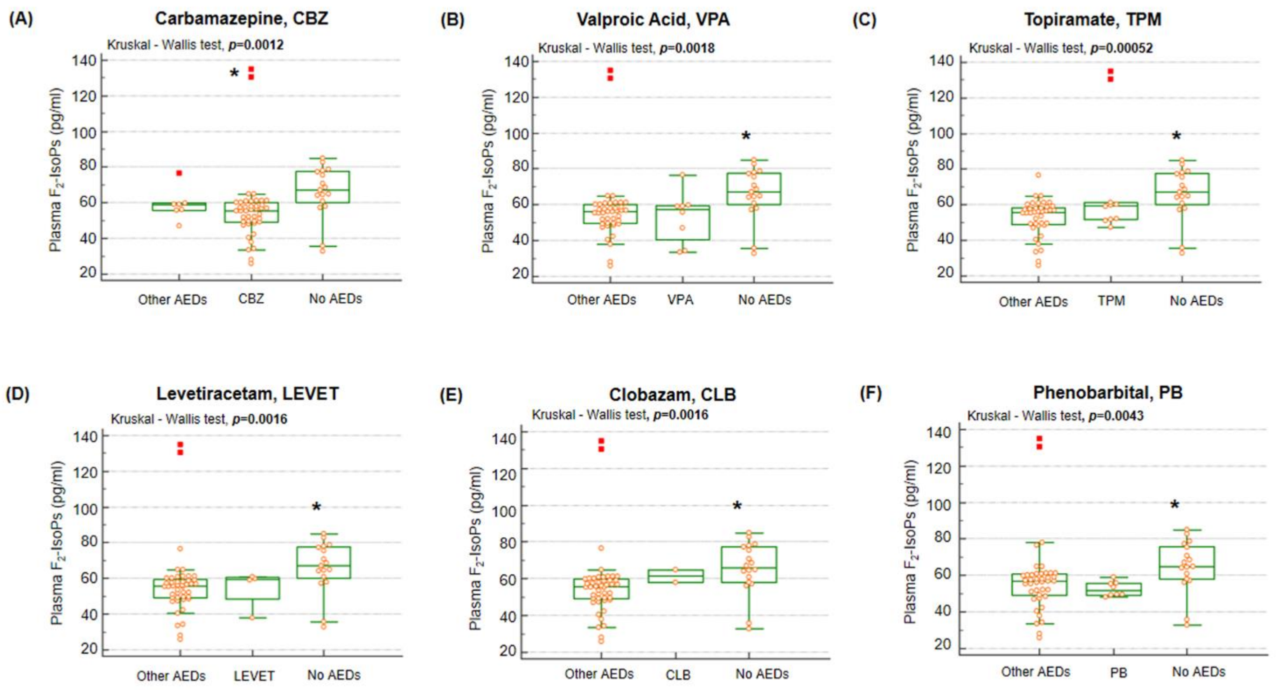

Supplementary Figure S10. Plasma F_2-IsoP levels as a function of antiepileptic drug therapy in the examined RTT population (n=66) **(A-F)**. **(A)** CBZ treatment. *Post-hoc* analysis (Conover test): * $p < 0.05$ *vs*. no AED group. **(B)** VPA treatment. Post-hoc analysis (Conover test): * $p<0.05$ *vs*. VPA group and other AED group. **(C)** TPM treatment. *Post-hoc* analysis (Conover test): * $p < 0.05$ *vs*. other AED group. **(D)** LEVET treatment. *Post-hoc* analysis (Conover test): * $p < 0.05$ *vs*. other AED group. **(E)** CLB treatment. *Post-hoc* analysis (Conover test): * $p < 0.05$ *vs*. other AED group. **(F)** PB treatment. *Post-hoc* analysis (Conover test): * $p < 0.05$ *vs*. PB group and other AED group. Plasma F_2-IsoPs: plasma F_2-isoprostanes. CBZ: carbamazepine. VPA: valproic acid. TPM: topiramate. LEVET: levetiracetam. CLB: clobazam. PB: phenobarbital. Data are shown as box- and whisker-plots. Red rectangles indicate outlier data points.
